# Direct electrical stimulation of the human amygdala enhances recognition memory for objects but not scenes

**DOI:** 10.1038/s42003-026-10270-4

**Published:** 2026-05-22

**Authors:** Krista L. Wahlstrom, Justin M. Campbell, Martina K. Hollearn, James Swift, Markus Adamek, Gansheng Tan, Lou Blanpain, Tao Xie, Tyler Davis, Peter Brunner, Stephan B. Hamann, Amir Arain, Lawrence N. Eisenman, John D. Rolston, Shervin Rahimpour, Joseph R. Manns, Jon T. Willie, Cory S. Inman

**Affiliations:** 1 Department of Psychology, University of Utah, Salt Lake City, UT USA; 2 Interdepartmental Program in Neuroscience, University of Utah, Salt Lake City, UT USA; 3 Department of Neurological Surgery, Washington University School of Medicine, St. Louis, MO USA; 4 National Center for Adaptive Neurotechnologies, St. Louis, MO USA; 5 Department of Neuroscience, Emory University, Atlanta, GA USA; 6 Department of Neurosurgery, University of Utah, Salt Lake City, UT USA; 7 Department of Psychology, Emory University, Atlanta, GA USA; 8 Department of Neurology, University of Utah, Salt Lake City, UT USA; 9 Department of Neurology, Washington University School of Medicine, St. Louis, MO USA; 10 Department of Neurosurgery, Mass General Brigham, Harvard Medical School, Boston, MA USA; 11 Dell Medical School, The University of Texas at Austin, Austin, TX USA

**Keywords:** Cognitive neuroscience, Human behaviour, Long-term memory, Neurophysiology, Hippocampus

## Abstract

Research from studies using invasive neuroanatomy-inspired direct manipulations suggests the basolateral amygdala (BLA) mediates a generalized modulation of many different types of memory. In contrast, noninvasive indirect correlations suggest that specificity exists in how the BLA prioritizes experiences in memory. We used direct electrical stimulation of the BLA to investigate the specificity of the memory enhancement in the human brain. Patients undergoing intracranial monitoring via depth electrodes viewed object and scene images, half of which were followed by BLA stimulation. Stimulation enhanced long-term memory for object but not scene images. Furthermore, BLA stimulation elicited stronger evoked responses in the anterior vs. posterior medial temporal lobe (MTL), regions that preferentially process object and scene learning, respectively. These results suggest the BLA exerts an important influence over the specificity of what information is prioritized in memory, rather than a general enhancement of all memory, and provide insight into how BLA-MTL projections contribute to the dynamics of memory prioritization.

## Abstract (web summary)

Direct electrical stimulation of the human amygdala influences the specificity of what information is prioritized in memory and provides insight into how amygdala-MTL projections contribute to the dynamics of memory enhancement.

## Introduction

Decades of learning and memory research suggest the basolateral amygdala (BLA) plays an essential role in enhancing emotional memories^1–11^. However, there is uncertainty as to whether this enhancement is a generalized boost or prioritizes some information over others. Studies using direct perturbations suggest the BLA mediates a causal influence on many different types of memory more generally^12–15^. In contrast, studies using indirect and correlational approaches propose that specificity exists for how the BLA prioritizes experiences in memory^16,17^. A limitation of this prior work is that numerous variables, including attention and arousal, covary with putative engagement of the BLA, making interpretation of the findings difficult. To circumvent these challenges, some studies in both rodents and humans have focused on direct manipulations of the BLA in the post-training memory consolidation period to dissociate learning effects from performance effects. However, to our knowledge, no prior study has used direct manipulations in this manner to investigate whether the human BLA prioritizes certain kinds of memories over others.

Increasing evidence in both humans and rodents suggests that BLA modulation of memory likely occurs via BLA interactions with other brain regions, including structures in the medial temporal lobe (MTL)^18–21^. Furthermore, research on human memory and direct perturbations of the BLA in rodents demonstrates the potential of BLA stimulation to interrogate these downstream connections^2–6,12,19–28^. For example, prior work in rodents suggests that memory-enhancing electrical stimulation of the BLA induces theta-modulated gamma oscillations in the hippocampus^29^, an oscillatory state previously correlated with good memory^30,31^. Similarly, recent work in humans found that direct electrical stimulation of the BLA enhances declarative memory, and this memory enhancement is marked by interactions between the BLA and hippocampus^20^. Together, these results from both rodent and human studies show that BLA modulation can induce specific memory-related signals in downstream regions. More specifically, direct activation of the BLA can enhance memory by modulating plasticity in the hippocampus.

Prior neuroanatomy studies using fluorescent tract tracing in both rodents and non-human primates suggest that BLA inputs to the hippocampus and adjacent MTL structures exhibit a pattern that approximates an anterior-to-posterior gradient of stronger to weaker projections^32,33^. Specifically, the BLA sends more dense excitatory projections to the anterior hippocampus (aHC; the homolog of ventral hippocampus in rodents) and perirhinal cortex compared to the posterior hippocampus (pHC; dorsal hippocampus in rodents) and parahippocampal cortex^32,33^. Evidence from rats, humans, and non-human primates suggests that this anterior-to-posterior pattern of BLA connectivity parallels a functional trend of separation of non-spatial object information from that about spatial contexts^34–37^. More specifically, a large body of literature indicates that the aHC/perirhinal cortex is essential for recognition memory for individual items, such as images of objects^38,39^, whereas the pHC mediates memory for images of places such as outdoor scenes^40^. The rodent hippocampus has a clear septal-to-temporal (posterior-to-anterior in primates) gradient for more to less spatial information, and results from humans suggest a parallel gradient^35,41^. Moreover, although the relevance of the long axis of the hippocampus is largely defined by anatomy, there is much parallelism from the lens of emotional memory studies in humans. For instance, activity in the amygdala and the MTL memory regions is strongly correlated, and activity in anterior regions predicts memory for emotional items^42,43^. Consequently, the BLA appears to be anatomically situated to modulate memory for objects more so than memory for discrete scenes or spatial contexts. Nevertheless, prior studies have not yet investigated whether and how the BLA modulates memory for multiple types of memory in the same experiment. Thus, the premise that the BLA prioritizes some kinds of memories over others has not been directly tested.

Movement towards novel therapeutics designed to emulate endogenous mechanisms of memory enhancement requires an understanding of the basic science of normal memory function. The present study, therefore, sought to test whether directly engaging the amygdala results in a generalized modulation of memory or differentially prioritizes memory processes for objects versus scenes—types of information processed by distinct memory systems—through a more specific influence on memory. We tested whether brief electrical stimulation applied directly to the human BLA could enhance recognition memory for specific images of neutral objects or scenes without eliciting an emotional experience. Eight patients with depth electrodes placed in the amygdala, hippocampus, and other frontal-temporal lobe targets for clinical purposes performed a recognition-memory task for object and scene images. During encoding, half of the images were immediately followed by a 1-s low-amplitude (0.5–1 mA) theta-modulated gamma frequency (theta-burst) electrical stimulation to the amygdala, and 1 day later, participants were given a retrieval test. Amygdala stimulation at this low amplitude reliably improved later object-recognition memory without eliciting emotional responses and had no effect on scene-recognition memory. These results highlight the utility of stimulation to initiate endogenous memory prioritization processes in the absence of emotional input. Furthermore, while prior work suggests BLA stimulation enhances object memory^20,21^, no prior studies exist in which direct manipulations of the BLA were shown to improve one type of memory but not another. Thus, the present study is the first to investigate multiple memory types in the same experiment, and therefore, is the first to suggest that stimulation of the amygdala has the capacity to prioritize enhancement of specific declarative memories over others rather than a general enhancement of all memory.

To investigate the neural substrates underlying BLA stimulation and the prioritization of object memory, we also considered the relationship between the degree of BLA-hippocampal connectivity and the resulting memory effect. Effective connectivity can be used to determine the causal influence between regions in the human brain^44–47^. Recent work using effective connectivity to map human brain networks shows that single-pulse intracranial stimulation triggers a local electrical response at the stimulation site, as well as evoked responses (single pulse evoked potentials; SPEP) at downstream locations, in proportion to the strength of the effective connection between the two locations^48,49^. The magnitude of the SPEP provides a measure of connectivity that is correlated with both structural^50^ and functional^51,52^ connectivity among brain regions, with sufficient resolution to infer the direction of information flow between two regions^44^. Importantly, where theta-burst stimulation is useful for modulating memory through the synchronization of neural activity^21^, using this stimulation pattern to probe simple network connectivity relationships is challenging in part due to confounding simultaneous stimulation artifacts. Single-pulse stimulation is the standard validated approach for assessing structural and effective connectivity^44^. Therefore, we investigated whether and how single-pulse electrical manipulations of the human BLA differentially influence the amplitude of evoked responses in the aHC and pHC as a measure of BLA-MTL connectivity in the same participants who completed the objects and scenes recognition memory task. We found that electrical stimulation of the BLA reveals stronger effective connectivity with the aHC networks important for object memory compared to the pHC networks important for scene memory. These results are the first to use direct brain stimulation and measures of effective connectivity to provide a potential circuit mechanism by which the amygdala preferentially enhances object memory compared to scene memory in humans. Moreover, our findings suggest the BLA mediates a more specific memory enhancement rather than a generalizable influence on memory, at least in part, due to its projections to the MTL.

## Results

**Fig. 1:**
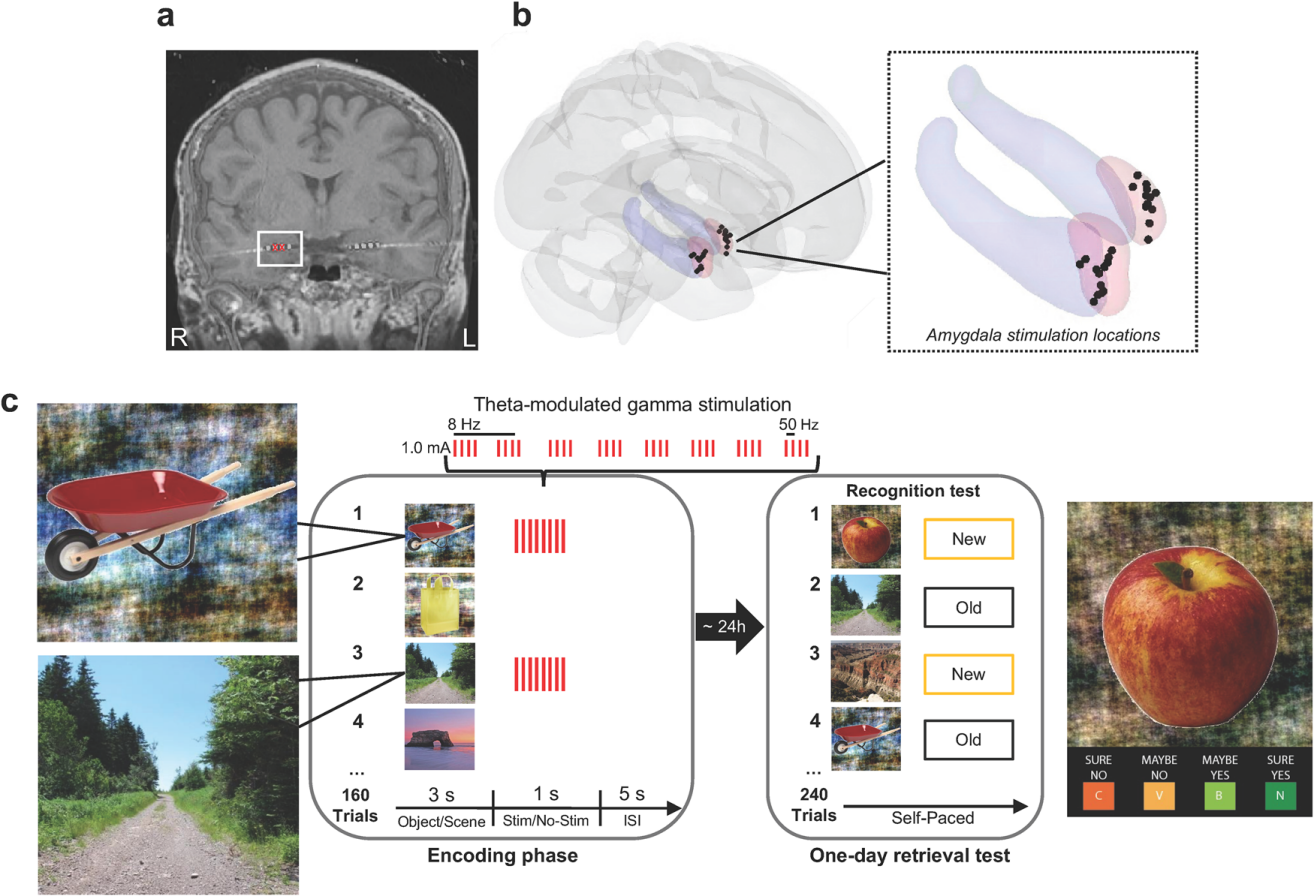
The procedure used to stimulate the human amygdala and to test recognition memory. **a** A representative postoperative coronal MRI/CT showing electrode contacts in the BLA (white square). The red Xs denote the bipolar pair of contacts stimulated in this example. **b** Brain template in Montreal Neurological Institute (MNI) space on which all amygdala stimulation electrodes have been plotted. The amygdala is highlighted in red, and the hippocampus is plotted in blue for reference. **c** Schematic of the recognition-memory task in which the BLA was stimulated (1-s stimulation pulse sequence; each pulse = 500 μs biphasic square wave; pulse frequency = 50 Hz; train frequency = 8 Hz) after the presentation of half of the images in the encoding phase, and recognition memory was tested for all images 1 day after the encoding phase. Example object and scene images (left). The object images were presented on scrambled backgrounds that were created to preserve the overall luminance, hue, spatial frequency, and shape between object and scene images (see Supplementary Material for more details on the stimuli). Example retrieval test image with the response options presented for each image (right).

The influence of amygdala stimulation on recognition-memory performance for neutral object and scene images was assessed in eight human participants across 14 different memory encoding/retrieval sessions (see Fig. 1, Supplementary Fig. S5, Supplementary Table S3, and Supplementary Table S1 for information on electrode locations and patient demographics). Figure 1c illustrates the recognition-memory procedure and depicts the 1 s of low-amplitude electrical stimulation (eight trains of 50 Hz, 0.5–1.0 mA biphasic pulses) that were delivered to the amygdala at the offset of image presentation for a randomly selected half of the object and scene images during the encoding phase of the task. Memory performance was tested 1 day later. Figure 1a shows a representative postoperative coronal CT/MRI depicting electrode contacts in the amygdala for one patient. Figure 1b represents all bipolar amygdala stimulation locations across all subjects plotted in MNI space.

### Electrical stimulation of the BLA enhances object memory but not scene memory

**Fig. 2:**
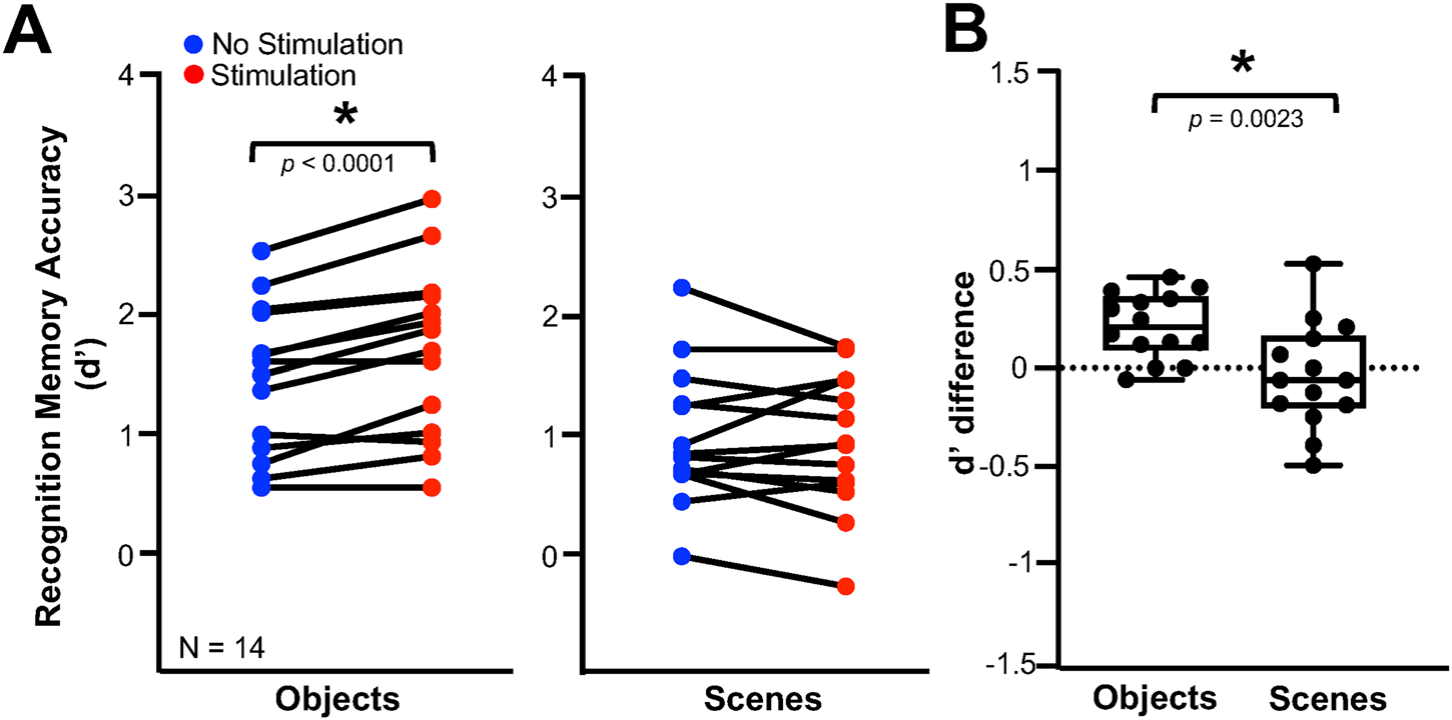
Brief electrical stimulation of the BLA in humans enhanced subsequent memory for objects but not scenes. **A** Recognition-memory performance for each of the 14 sessions across eight patients plotted as a discriminability index (*d*’) for object and scene images. **B** Recognition-memory performance plotted for each session as the difference in *d*’ in the stimulation and no-stimulation conditions (scatter plots in **A**). Overlaid box-and-whisker plots show the median, range, and interquartile range for each condition. *p < 0.05 compared with control images.

Figure 2A shows the memory outcomes as a standard discriminability index (*d*’), a typical approach for representing memory performance on a recognition task that accounts for an individual’s propensity to guess (for individual Hit and False Alarm rates see Supplementary Fig. S4). Results indicate that brief stimulation of the BLA during the encoding phase enhanced object-recognition memory relative to control (no-stimulation) object images the next day [*F*_(1,26)_ = 24.67, *p* < 0.0001]. By comparison, stimulation did not affect scene-recognition memory performance [*F*_(1,26)_ = 0.28, *p* = 0.60]. A standard 2 × 2 model did not yield a significant interaction for objects and scenes [*F*_(1,52)_ = 1.63, *p* = 0.21]. While our statistical approach accounts for the random effects of patients and sessions (*d*’ ~ 1 + Stim*ObjectScene + (1|Patient) + (1|Patient:Session)), our relatively small sample size prevents us from including the random effects variability of stimulation on object and scene memory that might vary across patients and/or sessions (i.e., (Stim*ObjectScene|Patient) + (Stim*ObjectScene|Patient:Session)). Thus, we next analyzed more explicitly whether the magnitude of the amygdala-mediated memory enhancement was greater for objects versus scenes. Figure 2B shows the recognition-memory performance plotted for each session as the difference in *d*’ between the stimulation and no-stimulation conditions for objects and scenes. Object images had a higher *d*’ difference score on average compared to scene images [objects vs. scenes *d*′ difference: mean(SEM) = 0.23(0.048) vs. −0.040(0.077), *F*_(1,26)_ = 11.45, *p* = 0.0023]. (See Supplementary Table S4 for patient-specific recognition-memory scores; See Supplementary Fig. S6 for memory performance separated by stimulation hemisphere and location.) Supplementary Fig. S8 shows the relationship between object and scene *d*’ difference scores. A Pearson correlation revealed no significant linear association between the stimulation-mediated object memory effect and the stimulation-mediated scene memory effect [*r*(12) = 0.11, *p* = 0.72, *R*² = 0.01, 95% CI [−0.45, 0.60]].

### BLA stimulation does not influence memory confidence

In the present recognition-memory task, patients were not only asked to mark images as “old” or “new,” but also how certain they were in their response and whether they had a conscious recollection of the images (“sure”) or simply knew they had been encountered before on some other basis (“maybe”). Supplementary Fig. S3 represents the analysis of these certainty judgments and shows that patients indicated they were “sure” more often for stimulated versus non-stimulated object images [*F*_(1,26)_ = 4.26, *p* = 0.049]. However, patients also indicated they had “maybe” encountered an image before more often for stimulated versus non-stimulated objects [*F*_(1,26)_ = 8.90, *p* = 0.0061]. This implies our main effect in Fig. 2 is the same whether we restrict our analyses to certain or uncertain responses, suggesting BLA stimulation enhances overall memory strength.

Supplementary Fig. S7 shows the receiver operating characteristic (ROC) curves for stimulated and non-stimulated object and scenes images. The probability of correctly recognizing an old image (Hit) and the probability of incorrectly identifying a new image as a target (False Alarm) are plotted across varied certainty judgments for each patient/session. Area under the curve (AUC) is a neutral measure of an ROC and a reliable measure of memory performance. Our results illustrate significantly higher AUCs for stimulated object images compared to non-stimulated object images [*F*_(1,26)_ = 10.71, *p* = 0.0030]. Stimulation did not affect AUCs for scene images [*F*_(1,26)_ = 0.09, *p* = 0.77]. These results are consistent with those shown in Fig. 2 and further suggest BLA stimulation enhances overall memory accuracy for object images.

### BLA stimulation enhances memory in the absence of subjective awareness

**Fig. 3:**
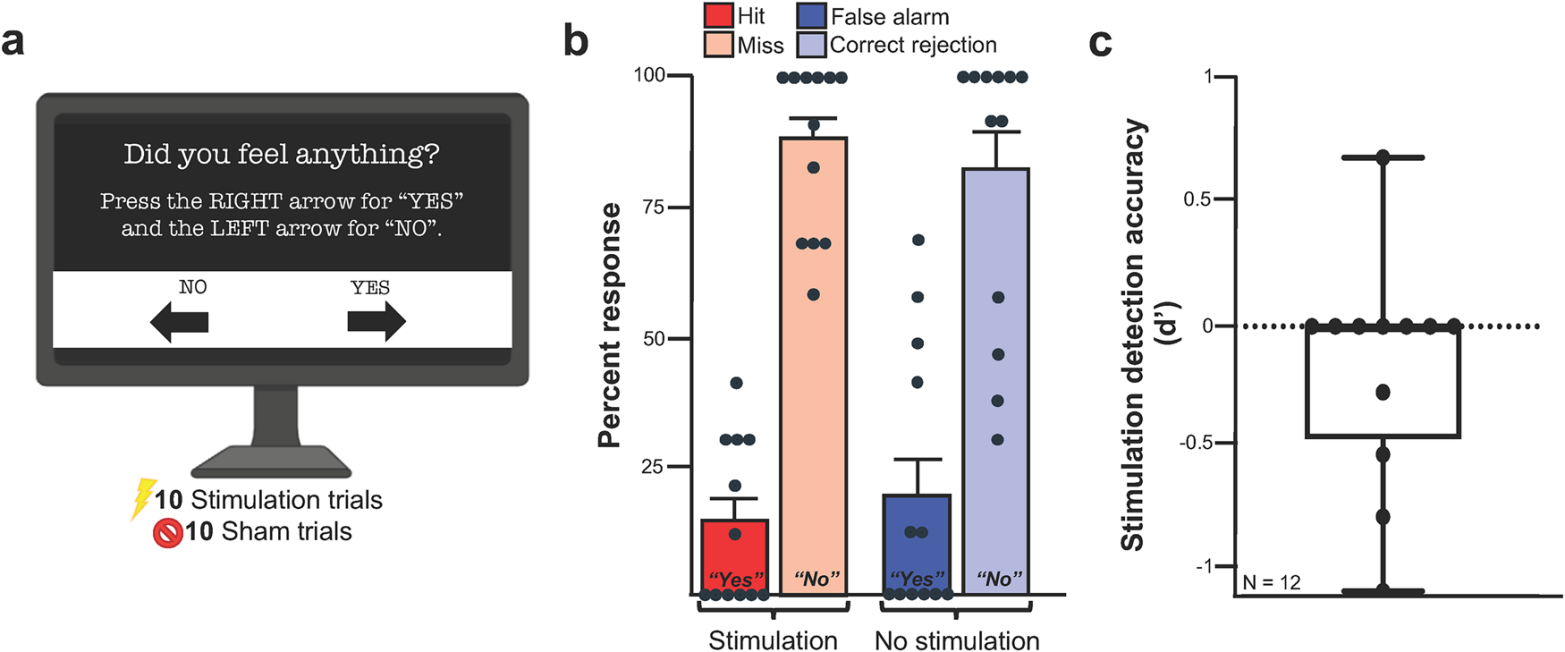
Participants could not reliably detect the presence of amygdala stimulation. **a** Illustration of the stimulation awareness test. Patients were given 10 amygdala stimulation trials and 10 sham/no-stimulation trials and were asked to report any sensations following each trial. Patients were blind to whether the trials were stimulation or sham trials. **b** Reported responses of patients when they were asked in stimulation awareness testing whether they felt any sensation of stimulation. Hits (correct “Yes” response for a stimulation trial), Misses (incorrect “No” response for a stimulation trial), False Alarms (incorrect “Yes” response for a no-stimulation trial), and Correct Rejections (correct “No” response for a no-stimulation trial). **c** Stimulation detection ability for each patient plotted as discriminability index (*d*’), incorporating the normalized hit and false alarm rates from (**b**). The one patient that appears to reliably detect the stimulation (with a *d*’ value of 0.62) had a Hit rate of 30%, False Alarm rate of 10%, Miss rate of 70%, and Correct Rejection rate of 90%.

Figure 3 shows the results of a subsequent stimulation awareness test in which participants were asked to indicate whether amygdala stimulation had been delivered across 10 BLA stimulation and 10 sham-stimulation trials that were presented in a random order. Patients’ responses indicate they were unable to reliably discriminate if or when any stimulation was being delivered (Fig. 3c) [*t*_(11)_ = 1.24, *p* = 0.24]. These results replicate those observed in prior work examining the effects of BLA stimulation on subjective awareness^20^. Furthermore, no patient reported emotional responses associated with the 80 stimulation trials during the memory encoding phase of the recognition-memory task. Thus, the amygdala stimulation used in the present study was capable of enhancing 1-day object recognition memory in the absence of any subjective emotional response.

### BLA stimulation does not influence reaction time

No differences in reaction times during the 1-day retrieval test were observed between stimulation and no-stimulation control images, suggesting that amygdala-mediated memory enhancement did not produce reduced reaction times in subsequent testing (Supplementary Fig. S2B). However, irrespective of stimulation and no-stimulation conditions, reaction times were significantly longer for scene images compared to object images during the 1-day retrieval test (Supplementary Fig. S2) [scenes vs. objects reaction time: mean(SEM) = 2.84(0.23) vs. 2.22(0.14), *t* (27) = 4.76, *p* < 0.0001].

### Single-pulse electrical stimulation reveals stronger effective connectivity between the BLA and aHC compared to the BLA and pHC

**Fig. 4:**
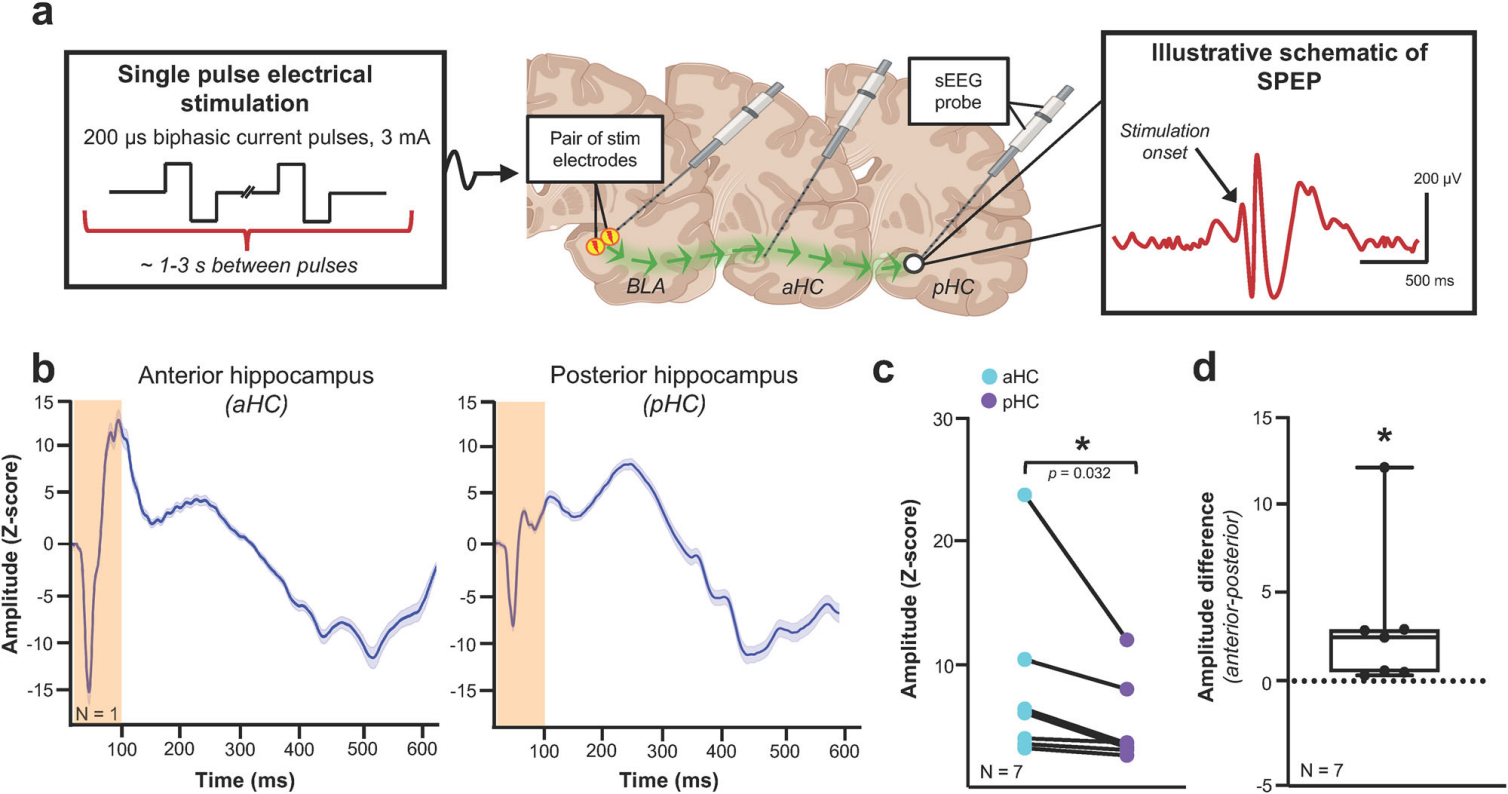
Single-pulse electrical stimulation of the BLA induces higher magnitude responses in the anterior compared to the posterior hippocampus. **a** Schematic of the stimulation procedure used to deliver high-amplitude single-pulse stimulation to the BLA and record evoked potential responses in the hippocampus (Created in BioRender. Wahlstrom, K. (2026) https://BioRender.com/972nh5h). **b** Example of a trial-averaged single-pulse evoked potential (SPEP) recorded from one anterior hippocampus channel (left) and one posterior hippocampus channel (right) from one patient. Stimulation occurred at time = 0 ms. Shading around each SPEP trace represents the SEM for the 30 trials of stimulation delivered. The orange shaded area represents the time window used to extract the amplitude. **c** The maximum stimulation response amplitude recorded in the 100 ms poststimulation averaged across all anterior and posterior hippocampus electrodes for a subset of patients/stimulation locations included in the recognition-memory procedure. **d** The SPEP amplitudes plotted as the difference between the anterior and posterior responses (scatter plots in **c**). *p < 0.05.

Figure 4a illustrates the stimulation procedure used to examine effective connectivity between the BLA and the hippocampus. Standard amplitude (3 mA) single-pulse electrical stimulation was delivered to the BLA while evoked responses were recorded in the aHC and pHC. (No patient reported feeling the presence of this stimulation.) Fig. 4b shows a representative example of a trial-averaged waveform in the aHC and pHC for one patient. Figure 4c shows the trial-averaged voltage peak amplitudes of the stimulation-evoked SPEPs averaged across all aHC electrode contacts and pHC contacts for each stimulation session. Figure 4d shows the SPEP amplitudes plotted for each session as the difference in amplitude between the aHC and pHC. Stimulation revealed significantly stronger effective connectivity, as measured by the magnitude of the evoked responses, in the aHC networks important for object memory compared to the pHC networks important for scene memory [*F*_(1,12)_ = 5.87, *p* = 0.032; Effect size (fixed effects coefficient for *Z*-scored amplitudes) = 0.53]. Together, these results suggest stronger effective connectivity between the BLA and aHC compared to the BLA and pHC, providing a potential mechanism by which BLA stimulation enhances object memory over scene memory.

## Discussion

The present study aimed to determine whether direct manipulation of the BLA would reveal the specificity of the memory enhancement for different types of visual stimuli. Our results suggest that direct electrical stimulation of the human amygdala reliably improves long-term recognition memory for images of neutral objects, but not for scenes, types of information processed by distinct memory systems in the anterior and posterior MTL, respectively. Furthermore, this memory enhancement occurred in the absence of a subjective emotional response, indicating a causal role for the amygdala in memory that is dissociable from emotional experience. Finally, our results provide evidence for stronger effective connectivity between the BLA and aHC compared to the BLA and pHC, suggesting the BLA mediates a more specific memory enhancement rather than a generalizable influence on memory, at least in part, due to its projections to the MTL.

Stimulation of the BLA enhanced object memory in an “event” specific (trial-specific) manner. Indeed, with amygdala-mediated memory enhancement, we not only observe stimulus specificity for object memory but also temporally specific learning that is robust after a single trial and persists the following day. Specifically, no carryover effects of stimulation on memory were observed for control images that followed stimulation trials during the study phase (Supplementary Fig. S1). The lack of carryover indicates that the object memory enhancement was temporally specific. That is, objects in the stimulation condition were remembered better than control objects initially viewed only seconds (~7 s) apart. This temporal specificity implies that long-lasting effects, such as increases in blood hormone levels, are unlikely to explain the observed memory enhancement. Furthermore, this temporal specificity is consistent with evidence that BLA activity in the consolidation period immediately following an experience is important for later retrieval of that experience^12^. A possible mechanism for the observed temporally specific amygdala-mediated memory enhancement involves changes in synaptic strength following an experience. Evidence suggests change in synapse function is involved in memory storage, and that the BLA in particular modulates the molecular processes that occur during the consolidation period^53^. Prior work shows that the BLA modulates hippocampal synaptic plasticity and modulates the expression of plasticity-associated proteins like ARC in a memory-dependent fashion^54,55^. Furthermore, memory-influencing BLA stimulation enhances ARC levels in the hippocampus^55^, providing a possible molecular mechanism by which our direct electrical stimulation of the BLA enhances object memory in a temporally precise manner.

The present study suggests that a possible circuit mechanism by which BLA stimulation preferentially enhances object memory compared to scene memory involves increased effective connectivity between the BLA and aHC, compared to the BLA and pHC. Specifically, single-pulse electrical stimulation of the BLA resulted in higher amplitude SPEPs in the aHC versus pHC, which is consistent with our finding that direct electrical stimulation of the BLA enhances object memory but not scene memory. Moreover, these results are unlikely to be due to spatial proximity alone, as prior work suggests SPEP amplitude is not just a monotonic function of distance from the stimulation site^56–58^. Importantly, prior work suggests SPEP amplitude is better predicted by white-matter tract properties than by simple Euclidean distance^58^. That is, distance alone is unlikely to account for the network topographies we report. Overall, our results suggest the BLA influences the specificity of what information is prioritized in memory rather than a general enhancement of all memory, and that BLA interactions with the hippocampus and related MTL structures may be important for this specificity.

Studies using direct perturbations suggest the BLA wields a generalizable influence on memory^12–15^. In contrast, studies using more indirect interrogations propose that specificity exists for which experiences are prioritized in memory^16,17^. To our knowledge, no prior studies exist in which direct manipulations of the BLA were shown to improve one type of memory but not another. Critically, prior studies did not investigate multiple memory types in the same experiment. In contrast, the emotional memory literature is replete with examples of how emotion enhances memory in some instances and impairs or has no effect on memory in other cases^16,59^. One possible explanation for the inconsistencies in the emotional memory literature is the imprecision of the experimental manipulations available to researchers, combined with the numerous indirect routes by which emotion can influence memory. However, the present study uses direct manipulations to suggest that BLA stimulation has the capacity to influence certain memories over others in the absence of subjective awareness or emotional response. The projections from the BLA to the hippocampus are one potential source for how certain memories are prioritized over others.

To further understand the BLA’s preferential effects on object learning, it is also critical to investigate the direct relationship between the areas stimulated and the resulting behavioral effect. Neurostimulation modeling is an increasingly popular tool for predicting the effects of stimulation on neural tissue in the human brain and relating these effects to meaningful behavioral changes^60^. One aspect of neurostimulation modeling involves examining the volume of neural tissue activated (VTA)^61^. VTA estimates incorporate the electric field distribution to quantify the location and extent of neuronal activation caused during electrical stimulation^60^. Furthermore, recent developments in neurostimulation modeling approaches integrate DTI neuroimaging data to examine the underlying white matter anatomy and structural connectivity to estimate the effects of electrical stimulation directly on the prevalent tracts^62,63^. Numerous clinical studies have recently implemented neurostimulation modeling techniques in the human brain to map the local and network effects of deep brain stimulation (DBS)^64–66^. For example, DBS targeting the fornix may enhance cognitive function in patients with Alzheimer’s disease, and neurostimulation modeling approaches suggest that variable outcome is correlated to the effects of focal electric fields on specific white matter tracts traversing the stimulation volumes^64^. Thus, neurostimulation modeling is a powerful approach for examining the intersection between the VTA and estimated neural pathway activation in studies that use direct electrical stimulation of the human brain. Future work will investigate whether and how stimulation of the human BLA differentially activates BLA-aHC and BLA-pHC structural connections and does so in a manner related to memory for objects, scenes, and other types of stimuli.

Our study raises several methodological and interpretive considerations. First, as in any study examining the effects of direct electrical stimulation in subjects undergoing intracranial monitoring, our study necessarily consisted of patients with epilepsy rather than healthy participants. Intracranial monitoring samples extensive territories of the brain representing normal and pathological tissue, to define the boundaries of each patient’s seizure focus accurately^67,68^. Although specific areas of each patient’s brain were presumed to be functioning abnormally, we accounted for the clinical heterogeneity across patients by using a within-subject design, comparing each patient’s memory performance in the stimulated versus non-stimulated conditions. Memory-enhancement effects were observed regardless of the differences in seizure foci across patients, including five sessions in which the amygdala and 10 sessions in which the hippocampus ipsilateral to BLA stimulation were implicated in the seizure focus (Supplementary Table S2). Comparable memory-enhancement results resembled those from prior work in humans^20^ and several prior studies in rats without seizures^19,21,23^, suggesting that our findings are unrelated to epilepsy. However, this remains a limitation to studies involving human intracranial electroencephalography.

Second, while our results indicate an effect for BLA stimulation on object memory but not scene memory, a standard 2 × 2 model did not yield a significant interaction for objects and scenes. Our linear mixed effects model accounts for the tangential random effects variability of patients and sessions (Dprime ~ 1 + Stim*ObjectScene + (1|Patient) + (1|Patient:Session)). However, given the constraints of collecting data from patients in a clinical setting, our dataset is small, necessitating relatively few model parameters. Thus, with the present statistical approach, we are unable to account for the random effects of stimulation on object and scene memory that might vary across patients and/or sessions because the model will not converge on a viable solution for this most fully parameterized model (Dprime ~ 1 + Stim*ObjectScene + (Stim*ObjectScene|Patient) + (Stim*ObjectScene|Patient:Session)). More specifically, the resulting covariance matrix is not positive definite, due to more model parameters than the data can support. However, in principle, a 2 × 2 interaction is a difference of differences. Thus, the significant result in Fig. 2B showing the recognition-memory performance plotted for each session as the difference in *d*’ between the stimulation and no-stimulation conditions for objects and scenes more explicitly illustrates the magnitude of the amygdala-mediated memory enhancement for objects versus scenes.

Third, the present findings observed variability in the baseline memory performance for objects versus scenes. Specifically, the recognition-memory accuracy for non-stimulated scene images was lower than that of non-stimulated object images [*F*_(1,26)_ = 6.06, *p* = 0.021], likely due in part to the differences in nameability^69^ between objects and scenes, raising the possibility that the lack of memory enhancement for scene images was due to a floor/ceiling effect. Although based on the numerical averages for recognition-memory accuracy, there was still sufficient statistical room for a scene memory enhancement to be observed. Moreover, prior learning and memory studies suggest that weaker baseline memory performance tends to show the largest stimulation-mediated memory improvement^20,70^, thus scenes had a greater advantage for memory enhancement. Nonetheless, it is difficult to be certain that a neurobiological floor effect, in which the memory itself could not be enhanced, did not occur. Moreover, the lack of memory enhancement for scene images also raises the possibility of insufficient engagement of pHC-related pathways. While the present study suggests a single dissociation of BLA projections to the aHC, future studies targeting alternative modulatory regions in the MTL to engage the pHC are of interest. Fourth, given the constraints of collecting data from a rare patient population in a clinical setting, our dataset is small, and electrode coverage is sparse. Thus, the observed aHC-pHC effective connectivity results necessitate cautious interpretation. Similarly, given that our analyses were restricted to where electrodes were placed for clinical purposes, we were unable to sufficiently examine effective connectivity in the perirhinal cortex and parahippocampal cortex across all patients. Therefore, it remains unclear how these regions canonically associated with object and scene processing, respectively, respond to BLA stimulation.

Finally, the observed memory-enhancement effect for objects was reliable and significant with no effect on scene memory, in contrast to previous studies that implicated the BLA in spatial and contextual learning. Evidence in rodents suggests optogenetic stimulation of the BLA enhances spatial memory and engages the dorsal hippocampus^18,55^. However, this prior work targeted BLA projections to the medial entorhinal cortex, suggesting that pathway manipulations are important for BLA modulation of spatial memory. Although we are unable to deliver electrical stimulation to distinct BLA projections in the human brain, our stimulation more fully engages the widespread projections of the BLA, and our results suggest stimulation preferentially engages the denser anatomical projections from the BLA to the aHC object memory system. Furthermore, unlike the present experiment in which object and scene memory were tested in the same paradigm, to our knowledge, no human or rodent study to date has directly compared multiple memory types in a single experimental paradigm. Thus, task demands and interactions between memory systems may be contributing to the present results.

## Conclusions

We leveraged electrical stimulation to determine what one can uniquely learn from direct manipulation of the BLA in the human brain. Our results suggest BLA stimulation elicits no subjective emotional response but enhances long-term memory for object images, but not scene images. Furthermore, single pulse electrical stimulation of the BLA elicits higher magnitude evoked responses in the aHC compared to the pHC, suggesting a potential mechanism by which BLA stimulation enhances object memory but not scene memory. Our findings suggest the BLA mediates an important preference for what information is prioritized in memory, rather than a general enhancement of all memory, and that BLA interactions with the hippocampus may be important for this preference. These experiments provide critical knowledge regarding how human MTL network connectivity, rather than individual brain structures, influences memory consolidation. Moreover, these findings represent a significant advancement in the basic science of normal memory function and a step towards novel therapeutics designed to emulate endogenous mechanisms of amygdala-mediated memory enhancement.

## Methods

### Participants

Eight participants (five females, three males) with drug-resistant epilepsy volunteered and provided written informed consent for all study procedures (see Supplementary Material for inclusion/exclusion criteria). The study protocol was approved by both the University of Utah and Washington University, St. Louis, Institutional Review Boards. All ethical regulations relevant to human research participants were followed. Stereotactic depth electrodes were implanted into the brain parenchyma by neurosurgeons for the sole purpose of clinical seizure investigation.

### Brain stimulation and recording

Electrode contacts were localized using LeGUI^71^ or VERA^72^, semi-automated detection and localization platforms for intracranial electrodes, following coregistration of pre- and postoperative brain imaging (Fig. 1a). Theta-burst stimulation parameters replicated those used in prior rat studies^19,21,23^ and a human study^20^ that demonstrated amygdala-mediated memory enhancement and physiological neuromodulation. Specifically, bipolar stimulation was delivered unilaterally to the BLA in current-regulated, charge-balanced, cathode-first biphasic rectangular pulses at 1.0 mA (or 0.5 mA for three of the 14 sessions) for 1 s in eight trains of four pulses at 50 Hz (Fig. 1c). Single-pulse bipolar stimulation for the effective connectivity analyses was applied to the BLA at 3.0 mA with a 1–3 s interpulse interval (Fig. 4a; See Supplementary Table S3 for patient-specific stimulation locations). SPEPs were recorded at a sampling rate of 2000 Hz. Stimulation did not elicit seizure activity or afterdischarges in a thorough pre-test stimulation safety session conducted with a clinical epileptologist for each patient. Implanted electrodes were recorded continuously and supervised by the clinical electrophysiology team for monitoring of induced epileptiform activity or electrographic seizures.

### Behavioral tasks

#### Recognition-memory task procedure

During the encoding phase, each participant viewed 160 object and scene images (80 neutral object images and 80 outdoor scene images) one trial at a time (Fig. 1c) (see Supplementary material for additional details on the stimuli). The object and scene stimuli were randomly intermixed. Participants were asked to make a “like” or “dislike” judgment with respect to the image on each trial (or, in the case of three of the 14 sessions, a “memorable” or “not memorable” judgment) to orient and encourage participants to pay attention to each image. This difference is an evolution of the experimental task design, given that some participants noted confusion in comprehending the task instructions. However, all participants included in the present study confirmed a clear understanding of the task instructions regardless of which orienting task was used. A fixation screen was presented for a jittered duration of 0.5–1.5 s immediately before each image onset. Each image was presented for 3 s, and for a randomly selected half of the trials (stimulation condition), an offset of the image coincided with the onset of 1 s of stimulation to the BLA. Our randomization ensured there were equal numbers of object and scene images that were paired with stimulation and no stimulation. The interstimulus interval was 5 s. Memory was tested approximately 1 day later (mean = 22 h, range = 18–25 h delay) via a self-paced old/new recognition-memory test for all 160 images. Eighty new images of each type served as foils (new images only occurred during the 1-day retrieval test and were never associated with stimulation). Foil images were used to assess each participant’s propensity to guess (false alarm), and performance on these new images was used as an exclusion criterion for the experiment (see Supplementary Material for additional details on exclusion criteria). After making old/new judgments, participants also made sure/maybe certainty judgments. No electrical stimulation was delivered during the memory retrieval test. Some participants completed the memory task multiple times on different days with a new image set and a different amygdala stimulation location. Thus, across the eight participants, a total of 14 different testing sessions were conducted. (Linear mixed effects modeling was used to account for the multiple testing sessions within each patient. See Supplementary Material for additional details on the analysis procedure.)

#### Stimulation awareness test procedure

After the 1-day retrieval test, participants were administered 20 trials in which stimulation or sham stimulation (no current) was randomly delivered to the BLA (Fig. 3a). Participants were asked “Did you feel anything?” Stimulation to the BLA using the same parameters as during the memory task was delivered, and participants were blind to which trials were paired with stimulation.

### Electrophysiological data analysis

Analyses of electrophysiological data focused on evoked potentials recorded from the hippocampus in five of the eight total patients who had both anterior and posterior hippocampal electrode sampling for seven different BLA stimulation locations. Electrodes were bipolar re-referenced relative to nearest neighbors to account for volume conduction^49^. The data were filtered with a Butterworth bandpass filter (0.1–150 Hz). To attenuate 60 Hz electromagnetic noise in the evoked responses, 60 Hz and 120 Hz notch filters were applied. To analyze the SPEPs for the aHC and pHC, we created peri-stimulation epochs for each stimulation trial per region. Data were divided into epochs spanning −900 to −10 ms prestimulation and 10–900 ms poststimulation (with stimulation onset at time zero) for each stimulation trial^57^. We visually inspected the data and rejected trials with epileptiform spikes. For the remaining trials, voltages were normalized (*z*-scored) to the prestimulation baseline data segment per stimulation trial. We measured the normalized peak amplitude of the SPEP in the 100-ms poststimulation period^57^ and calculated the trial-averaged voltage peak amplitude. SPEPs may^72^ have long-lasting responses^73^; we studied the early response period, which has been related to structural features of network connectivity^50^. To control for individual differences, we compared the SPEP amplitude in the aHC to the SPEP amplitude in the pHC for each patient/stimulation location.

### Statistics and reproducibility

Linear mixed effects modeling was performed to account for the multiple testing sessions within each patient (fixed effects for stimulation condition, random effects for patient and session). See the Supplementary Information for details on the dependent variable used for each specific analysis.

## Supplementary information

Transparent Peer Review file

Supplementary Information

## Data Availability

The datasets generated are available through the NIMH Data Archive (collection ID 3688).

## Code availability

The code used to analyze the current study’s data is available on the INMAN Laboratory’s GitHub repository (https://github.com/INMANLab/BLAES_Objects-Scenes_Manuscript_Wahlstrom).
