# Supplementary material for: Direct electrical stimulation of the human amygdala enhances recognition memory for objects but not scenes
Source: Commun Biol. 2026 May 22;9:1100. doi: 10.1038/s42003-026-10270-4 (PMC13473522; doi:10.1038/s42003-026-10270-4)
Supplement: Supplementary file 1 [file 42003_2026_10270_MOESM1_ESM.pdf]

Peer Review File

Direct electrical stimulation of the human amygdala enhances recognition memory for objects but not scenes

Corresponding Author: Dr Krista Wahlstrom

This file contains all reviewer reports in order by version, followed by all author rebuttals in order by version.

Version 0:

Reviewer comments:

Reviewer #1

(Remarks to the Author)
The manuscript presents an intriguing and well-executed study examining the effects of direct electrical stimulation of the human basolateral amygdala (BLA) on recognition memory for objects and scenes. The combination of behavioral results and effective connectivity analyses is compelling, and the work addresses an important gap by testing multiple stimulus types within the same participants. The finding that low-amplitude theta-burst stimulation selectively enhances object memory, in the absence of subjective emotional experience, is potentially of high interest to the field.

However, several points should be addressed to strengthen the claims and improve clarity:

1. The manuscript reports a selective enhancement of object memory following BLA stimulation. Could the authors elaborate, with reference to prior literature such as Inman et al. (2018) and Bass & Manns (2015), on the precise mechanistic or conceptual advances this study offers relative to existing findings?

2. As all participants were individuals with drug-resistant epilepsy, how do the authors view the implications for generalizability to healthy populations, and have they considered additional controls or comparative data that might strengthen this claim?

3. The core claim of "selective" enhancement for object memory is not supported by a significant stimulation × stimulus-type interaction in the primary 2×2 analysis ($p=0.21$). Please clarify whether this interaction was a preregistered primary test. If so, discuss the implications of its non-significance more explicitly. If not, justify the use of post-hoc comparisons and difference scores, and report corresponding effect sizes and confidence intervals. A power analysis for detecting the interaction would also be valuable.

4. To substantiate the conclusion that the null effect for scenes reflects genuine stimulus-type specificity, how do the authors distinguish this from the possibility of insufficient engagement of pHC-related pathways or behavioural ceiling/floor constraints? Are follow-up studies planned to address this distinction?

5. The stronger BLA–aHC than BLA–pHC evoked responses are consistent with the behavioral pattern, but the small sample ($n=5$) and limited electrode coverage necessitate cautious interpretation. Please report effect sizes, individual data, and any correlation between aHC–pHC differences and behavioral enhancement at the session level. Additionally, detail preprocessing steps (e.g., filtering, artifact rejection) and justify the chosen 100 ms window.

6. Some sessions used "like/dislike" judgments, while others used "memorable/not memorable." Please justify this variation and clarify whether preliminary analyses confirmed no interaction between judgment type and stimulation effects. If possible, include this factor in the statistical models.

7. Minor typographical issues (e.g., "in the same the experiment," p. 10, line 360).

Reviewer #2

(Remarks to the Author)

Wahlstrom and colleagues present a manuscript characterizing how stimulation of the BLA influences memory for neutral objects and scenes. The authors show that BLA stimulation selectively enhances memory for objects, but not scenes. Moreover, they show that single-pulse stimulation of the BLA results in greater connectivity with anterior versus posterior hippocampus and propose that this could be the mechanism driving the content-specific effects. While there is a large body of research characterizing amygdala-MTL interactions during emotional memory, the current study builds to a very small literature looking at causal manipulations of this circuit. The study is strengthened by adding a layer of content specificity that will be of high interest for memory researchers. The experimental design is simple and appropriate for the questions at hand, and the analysis plan was executed nicely (although I did have a few clarifying questions). The results are clean and advance the literature on emotional memory, making these findings an important contribution to the field. Despite these strengths, there were a few weaknesses in the manuscript that decreased the overall impact of the paper but should be addressable in revision. These were centered on some of the theoretical framing/interpretation of the findings. I detail these concerns and a few others below.

1. I think the introduction in many ways provides an incomplete picture of the current field, and in many areas is too under-specified to understand the contributions of the current study. First, I really struggled with the vague concept of reconciling the neuroscience and psychology literature, as the authors never dive deep into either of these literatures to motivate that tension (also is there a tension?). Particularly the idea of “some memories over others” seemed extremely under-specified. Especially, because a ton of animal work shows disassociations of the BLA across different learning systems. Indeed, there is a rodent paper that shows that BLA lesions impair familiarity and not recollection (i.e., Farovik et al, 2011), which fits well with theoretical models of emotional memory. In this way, the proposed tension that this study is resolving seems a little superficial. To be clear, I think this study is extremely important, but it’s importance could be placed in a more nuanced conceptual framework that is better supported by the empirical literature. Second, I think more detail needs to be provided about the current state of long-axis differences in the MTL that has emerged from the neuroimaging literature. The motivation for long-axis was namely defined around anatomy, but there is a great deal of work showing parallelism from the lens of emotional memory studies in humans. Even an early paper by Dolcos, Labar, and Cabeza (2006) prominently highlights the long axis separation. Third, more background is needed on content specificity across the long-axis, and studies detailing these differences, and why the selection of object and scene stimuli were so important.

2. Could the authors provide more explanation of why theta-burst stimulation is bad for probing network connectivity? I think many people in the e-phys world know this, but I think this paper will be of broad interest. I was also a little confused, becuase the authors are on a paper that looks at connectivity during theta-birst stimulation. Is there something unique about the current recording approach that didn't allow this type of analysis. Similarly, couldn't the authors look at phase coherence across regions as they did in their earlier PNAS paper. I guess I need some more contextualization why these other analyses were not performed.

3. Do the authors have any electrodes in the cortical MTL, or is it entirely in the hippocampus. Much of the prior literature would suggest that the type of memory the authors are testing should/could be supported by PRC and PHC, but they only report on anterior/posterior hippocampus. If they do not have these recordings, I think this point should be detailed further in the discussion.

4. I was curious about how spatial proximity influences their connectivity measures? By definition would any region that is closer to the stimulation site show greater connectivity, or is there something unique to the AMG-aHIP circuit. Is there any way for the authors to show connectivity with a control region that is of the same relative distance?

5. Why did some runs include like/dislike and others memorability judgements? I am not too concerned about this because session was controlled for in the multi-level models, but it did sort of stick out in the methods section.

6. I greatly appreciated the stimulation awareness test procedure that was included in each session, however, I think just showing a negative finding does not appropriately control for this measure. Given the authors’ use of multi-level modeling, I think stimulation awareness should be placed into the model in a way that can vary across sessions.

7. I appreciated the authors showing the stimulation site from one participant, but is there a reason why they couldn’t show all of them on a single brain. What would also be nice is for the BLA ROI to be highlighted in that cross section to show the concordance with anatomy.

8. Could the authors provide more details on how they did there statistical testing within the linear mixed effects modeling framework. My curiosity was raised particularly by the fact that content*stimulation interaction did not come out, despite there being robust differences for objects and differences in the opposite direction for objects.

9. I thought the treatment of the confidence data was insufficient. I think the authors should either use an estimate that incorporates these measures (i.e., ROC analyses) or do an anova that includes both confidence ratings within the same model.

10. The authors are suggesting that there could be a relationship between their connectivity findings and memory findings, and I wonder if there is a missed opportunity to empirically test this. Can the authors plot the data on the strength of connectivity against the memory results? I know this may be too small for statistical testing, but it could still be interesting to visualize (if only in the response to reviews).

Reviewer #3

(Remarks to the Author)
The authors reported intracranial stimulation experiments in neurosurgical patients with depth electrodes implanted in the basolateral amygdala. They report a selective improvement in memory for objects, but not scenes, under stimulation compared to non-stimulation conditions. They also examined single-pulse evoked potentials in the hippocampus and argue that BLA stimulation elicited stronger responses in the anterior than in the posterior hippocampus. The authors interpret these results as evidence that the amygdala's influence on memory was not a generic enhancement but was biased toward a specific stimulus type.

Overall, the study addressed an important question about the specificity of neuromodulatory influences on human memory. The figures are appropriate and the supplemental information is useful. However, there are some issues that should be addressed in a revision.

1. Please clarify whether object images and scene images were intermixed randomly during encoding or presented in separate blocks. Additionally, confirm whether the number of stimulation trials was strictly balanced across object trials and scene trials. The Methods section states that "for a randomly selected half of the trials (stimulation condition), an offset of the image coincided with the onset of 1 s of stimulation", but it remains unclear whether this randomization ensured an equal number of stimulated trials for each stimulus category.

2. The reporting of the number of encoding trials is inconsistent across sections of the manuscript. The main text describes the task as consisting of 160 images (80 objects and 80 scenes), while the Supporting Information indicates that each participant viewed 180 images in total (80 neutral object images presented on scrambled backgrounds, 80 outdoor scene images, and 20 scrambled-only images). It would be helpful to clarify how the scrambled-only images were handled, including whether they were part of the encoding phase across all sessions and whether there was stimulation following any of them.

3. It would be useful to test whether the improvement in object memory depends on baseline memory performance. Do patients with better memory under no-stimulation conditions show larger or smaller improvements? Additionally, it would be helpful to examine whether there is a relationship between the effects of stimulation on object memory and scene memory across patients. Specifically, whether patients showing greater improvements in object memory also tend to show stimulation effects on scene memory?

4. The authors concluded that "stronger effective connectivity between the BLA and anterior hippocampus (aHC) compared to the posterior hippocampus (pHC) provides a potential mechanism by which BLA stimulation enhances object memory over scene memory" based on the assumption that aHC networks preferentially support object memory whereas pHC networks preferentially support scene memory. However, this key assumption is not directly tested in the present dataset. To support this interpretation, I encourage the authors to analyze hippocampal activity during encoding, comparing responses to object vs. scene stimuli in the aHC and pHC. For example, is activity greater in aHC than pHC for object encoding, and greater in pHC than aHC for scene encoding? This would provide more direct evidence of the proposed functional specialization in this specific task.

5. The idea that BLA–aHC connectivity accounts for the selective enhancement of object memory is intriguing but remains indirect. The authors could consider testing whether SPEP amplitudes in the aHC correlate with the magnitude of behavioral memory enhancement when stimulating the same BLA contact, which would provide a more direct link between physiology and behavior.

6. Previous rodent studies have demonstrated that the BLA plays a role in spatial and contextual learning. In contrast, the present results showed that BLA stimulation did not influence scene memory. Please discuss whether species differences, task demands (recognition vs. navigation paradigms), or stimulation parameters might account for this discrepancy.

7. Supplementary Table S3 shows that stimulation sites varied across sessions and patients, including differences in hemisphere and in medial versus lateral locations within the BLA. Please comment on whether the behavioral effects differed by hemisphere or by medial versus lateral stimulation contacts.
8. Please ensure that panel labels (lowercase a, b or uppercase A, B) match the figure legends in all supplementary figures.

Reviewer #4

(Remarks to the Author)
I co-reviewed this manuscript with one of the reviewers who provided the listed reports. This is part of the Communications Biology initiative to facilitate training in peer review and to provide appropriate recognition for Early Career Researchers who co-review manuscripts.

Version 1:

Reviewer comments:

Reviewer #1

(Remarks to the Author)
Reviewer Comments (Revised Manuscript)
I would like to thank the authors for the careful revision. The manuscript has clearly improved, particularly in the clarification of the methods, the additional analyses, and the more explicit discussion of limitations. I also think the overall question—whether BLA stimulation differentially modulates distinct types of memory—is both important and well-motivated, and the combination of intracranial stimulation with behavioral and connectivity measures is a real strength of the work.

I only have a few remaining comments, mostly related to interpretation and phrasing.

1. Framing of stimulus-specific (object vs. scene) effects
The enhancement of object memory under stimulation is convincing and nicely demonstrated. At the same time, as the authors acknowledge, the primary stimulation × stimulus-type interaction was not significant.

While I understand the rationale for focusing on the planned contrast, I think the current wording (e.g., “selective” or “prioritized” enhancement) may be a bit stronger than what the data can fully support. To me, the results are clearly consistent with a preferential effect on object memory, but do not yet definitively establish selectivity.
I would suggest slightly softening the language here to better match the statistical evidence.

2. Interpretation of effective connectivity results
The addition of the connectivity analysis is a nice extension and adds an interesting mechanistic angle to the paper. That said, I would still interpret this part somewhat cautiously.

As presented, these analyses are based on a smaller subset and are not directly linked to the behavioral effects. For that reason, it may be more appropriate to frame them as pointing to a possible or candidate circuit mechanism, rather than a definitive one.

Reviewer #2

(Remarks to the Author)
I thank the authors for their thoughtful reviews, and only have one minor concern. The authors note that they do not show an interaction, but they can use difference scores, due to the small sample size and multi-level modeling approach. I completely agree with the authors rationale, but I think some discussion of this limitation in statistical analyses should be addressed in the manuscript, if only to inform readers of their analytical approach.

Open Access This Peer Review File is licensed under a Creative Commons Attribution 4.0 International License, which permits use, sharing, adaptation, distribution and reproduction in any medium or format, as long as you give appropriate credit to the original author(s) and the source, provide a link to the Creative Commons license, and indicate if changes were made.

In cases where reviewers are anonymous, credit should be given to 'Anonymous Referee' and the source.
The images or other third party material in this Peer Review File are included in the article's Creative Commons license, unless indicated otherwise in a credit line to the material. If material is not included in the article's Creative Commons license and your intended use is not permitted by statutory regulation or exceeds the permitted use, you will need to obtain permission directly from the copyright holder.
To view a copy of this license, visit https://creativecommons.org/licenses/by/4.0/

We thank the reviewers for their thoughtful critiques and insights. Based on their suggestions, we have conducted additional analyses and revised the manuscript (text highlighted in blue). We have done our best to improve our manuscript by addressing their suggestions and have included justifications for our revisions in the responses to each comment below.

Below, for brevity and ease of review, we have described the essence of each reviewer's critique and kept our responses as succinct as possible. We also include direct quotes from revisions made to the main manuscript and supplementary materials which can be found below in red.

Reviewers' Comments

Reviewer #1:

Reviewer #1 notes many strengths of the manuscript, including compelling behavioral results and effective connectivity analyses that are potentially of high interest to the field. The reviewer notes the study is well-executed and addresses an important gap in the field by testing multiple stimulus types within the same participants. The reviewer had some criticisms, largely centered on how the study offers conceptual advances relative to the existing literature and how the statistical analyses were conducted.

1. Reviewer 1 requested elaboration on the specific conceptual advances the present study offers relative to prior literature from our group, such as Inman et al., 2018 and Bass & Manns, 2015.

RESPONSE: In addition to the mechanistic advances our effective connectivity analyses provide, as cited throughout the Introduction and Discussion, we now explicitly state on page 5 the importance of our study in providing a potential circuit mechanism by which the amygdala prioritizes memory.

"These results are the first to provide a potential circuit mechanism by which the amygdala prioritizes object memory over scene memory in humans."

Furthermore, on page 4, we highlight the importance of our study in investigating multiple memory types in the same experiment, and we contextualize our work in relation to the Inman et al., 2018 and Bass & Manns, 2015 studies to highlight the novelty of the present work.

"Furthermore, while prior work suggests BLA stimulation enhances object memory[20,21], no prior studies exist in which direct manipulations of the BLA were shown to improve one type of memory but not another. Thus, the present study is the first to investigate multiple memory types in the same experiment, and therefore, is the first to suggest that stimulation of the amygdala has the capacity to prioritize enhancement of specific declarative memories over others rather than a general enhancement of all memory."

2. Reviewer 1 suggested the authors provide comment on how the present findings generalize to healthy populations, given the study necessarily consisted of patients with epilepsy.

RESPONSE: The reviewer raises an important issue, one that we agree is a limitation of the present work. We now include additional statements on the methods used to continuously monitor the clinical electrophysiology data for exclusion of epileptiform activity which can be found in the Methods on page 13 and page 14.

"Implanted electrodes were recorded continuously and supervised by the clinical electrophysiology team for monitoring of induced epileptiform activity or electrographic seizures."

"We visually inspected the data and rejected trials with epileptiform spikes."

We also provide more clarity on the limitations of studies involving human intracranial electroencephalography which can be found in the Discussion on page 11.

"Memory-enhancement effects were observed regardless of the differences in seizure foci across patients, including five sessions in which the amygdala and 10 sessions in which the hippocampus ipsilateral to BLA stimulation were implicated in the seizure focus (Supplementary Table S2). Comparable memory-enhancement results resembled those from prior work in humans[20] and several prior studies in rats without seizures[19,21,69], suggesting that our findings are unrelated to epilepsy. However, this remains a limitation to studies involving human intracranial electroencephalography."

3. Reviewer 1 requested clarification on the core claim of selective memory enhancement for object memory but not scene memory when a significant interaction was not observed in the primary stimulation x stimulus-type analysis.

RESPONSE: We appreciate the reviewer's comment. The reviewer is correct that we did not observe a significant stimulation x stimulus-type interaction in our primary 2x2 analysis. However, we want to clarify that the contrast of stimulation-no stimulation differences between objects and scenes is not a post-hoc data mining analysis but is instead a pre-planned contrast that is the most specific way to test the main hypothesis of the experiment. Our statistical approach accounts for the tangential "random effects" variability of patients and sessions (Dprime ~ 1 + Stim*ObjectScene + (1|Patient) + (1|Patient:Session)). However, given the constraints of collecting data from relatively rare patients in a clinical setting, our dataset is small which necessitates relatively few model parameters. Thus, we are unable to account for the "random effects" of stimulation on object and scene memory that might vary across patients and/or sessions because the model will not converge on a viable solution for this most fully parameterized model (Dprime ~ 1 + Stim*ObjectScene + (Stim*ObjectScene|Patient) + (Stim*ObjectScene|Patient:Session)). More specifically, the resulting covariance matrix is not positive definite, due to more model parameters than the data can support. Unfortunately, the limitations of the model used in the present study prevent us from identifying the true trend that stimulation enhanced object memory but not scene memory.

However, in principle, a 2x2 interaction *is* a difference of differences. Thus, the significant result in Figure 2B showing the recognition-memory performance plotted for each session as the difference in d' between the stimulation and no-stimulation conditions for objects and scenes more explicitly illustrates the magnitude of the amygdala-mediated memory enhancement for objects versus scenes.

We thank the reviewer for raising this point and we now more explicitly address the limitations of our statistical approach in the Results on page 6.

*"A standard 2 x 2 model did not yield a significant interaction for objects and scenes [$F_{(1,52)}$ = 1.63, p = 0.21]. While our statistical approach accounts for the random effects of patients and sessions (d' ~ 1 + Stim*ObjectScene + (1|Patient) + (1|Patient:Session)), our relatively small sample size prevents us from including the random effects variability of stimulation on object and scene memory that might vary across patients and/or sessions (i.e. (Stim*ObjectScene|Patient) + (Stim*ObjectScene|Patient:Session)). Thus, we next analyzed more explicitly whether the magnitude of the amygdala-mediated memory enhancement was greater for objects versus scenes. Fig. 2B shows the recognition-memory performance plotted for each session as the difference in d' between the stimulation and no-stimulation conditions for objects and scenes."*

4. Reviewer 1 asked whether the lack of memory enhancement for scene images was due to a floor/ceiling effect or insufficient engagement of pHC-related pathways.

RESPONSE: We do not believe floor/ceiling effects are responsible for the current findings. Based on the numerical averages for recognition-memory accuracy, there was still sufficient statistical room for a scene memory enhancement to be observed. Moreover, prior learning and memory studies suggest that weaker baseline memory performance tends to show the largest stimulation-mediated memory improvement (see Jun et al, 2020 and Inman et al., 2018), thus scenes had a greater advantage for memory enhancement. Nonetheless, it is difficult to be certain that a neurobiological floor effect, in which the memory itself could not be enhanced, did not occur. Furthermore, the present study is admittedly a single dissociation as double dissociation studies are not currently feasible in human clinical populations. Future studies targeting alternative modulatory regions in the MTL to engage the pHC are of interest, and we hope our paradigm can be used in future work dissecting MTL pathways in the way the reviewer suggests. We make these limitations and future directions clearer in the Discussion on page 11 and page 12.

"...raising the possibility that the lack of memory enhancement for scene images was due to a floor/ceiling effect. Although based on the numerical averages for recognition-memory accuracy, there was still sufficient statistical room for a scene memory enhancement to be observed. Moreover, prior learning and memory studies suggest that weaker baseline memory performance tends to show the largest stimulation-mediated memory improvement[71,20], thus scenes had a greater advantage for memory enhancement. Nonetheless, it is difficult to be certain that a neurobiological floor effect, in which the memory itself could not be enhanced, did not occur. Moreover, the lack of memory enhancement for scene images also raises the possibility of insufficient engagement of pHC-related pathways. While the present study suggests a single

dissociation of BLA projections to the aHC, future studies targeting alternative modulatory regions in the MTL to engage the pHC are of interest."

5. Reviewer 1 suggested given our small sample size, we be more cautious in our interpretation of the observed BLA-aHC and BLA-pHC evoked responses and suggests providing an effect size for the effective connectivity analysis. Furthermore, all three reviewers were curious about any potential correlation between aHC-pHC effective connectivity differences and behavioral memory enhancement. Reviewer 1 also requested details of the preprocessing steps used for the effective connectivity electrophysiological data analysis.

RESPONSE: We now include a statement on how our sample size warrants caution and limits our interpretation of the effective connectivity results which can be found in the Discussion on page 11.

"Third, given the constraints of collecting data from a rare patient population in a clinical setting, our dataset is small and electrode coverage is sparse. Thus, the observed aHC-pHC effective connectivity results necessitate cautious interpretation."

There is, unfortunately, no consensus on how to best calculate effect sizes for linear mixed effects models. Our mixed effect model partitions the variance between fixed and random effects, thus, it is difficult to determine how much variance is captured by anterior vs. posterior hippocampus. However, the estimate of the fixed effect coefficient for the Z-scored amplitudes could be interpreted as the change in amplitude from anterior to posterior hippocampus. Therefore, we now provide this coefficient as our effect size for the effective connectivity analysis in the Results on page 9.

"Stimulation revealed significantly stronger effective connectivity, as measured by the magnitude of the evoked responses, in the aHC networks important for object memory compared to the pHC networks important for scene memory [$F_{(1,12)} = 5.87$, $p = 0.032$; Effect size (fixed effects coefficient for Z scored amplitudes) = 0.53]."

While all three reviewers agree our sample size is too small for statistical testing of a correlation between the effective connectivity results and the memory enhancement effects, we have provided a visualization of this correlation for the reviewers below. We had included the preprocessing details for the effective connectivity analyses in the supplemental material. These details have now been shifted from the supplement to the main text Methods section on page 13 to provide the appropriate details necessary for the reader without necessity of the supplement.

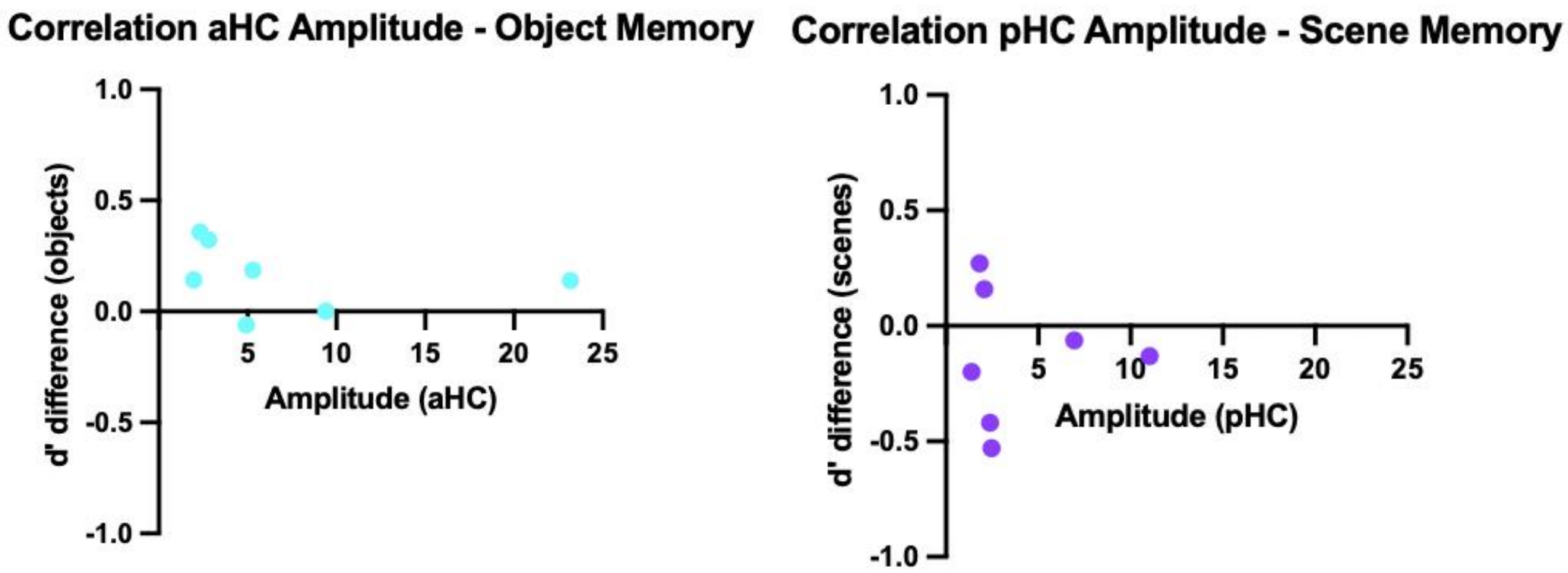

The anterior hippocampus (left) and posterior hippocampus (right) evoked response amplitudes from Figure 4C plotted against the recognition-memory performance scores (differences in d' in the stimulation and no-stimulation conditions) from Figure 2B for object and scenes, respectively.

6. Reviewer 1 and Reviewer 2 both noted the use of two different versions of the orienting task during encoding across participants, where for three of the 14 sessions a "memorable" or "not memorable" judgment was made with respect to the image on each trial, and in the case of 11 of the 14 sessions a "like" or "dislike" judgment. The reviewers request more clarity on this difference.

RESPONSE: We appreciate the reviewers' comments. An important note, as mentioned by Reviewer 2, is that although two different variations of the orienting task were used across the 14 encoding sessions, session is a random effect in our linear mixed effect model, thus this difference across sessions is already accounted for at the level of our statistical testing (Dprime ~ 1 + Stim*ObjectScene + (1|Patient) + (1|Patient:Session)). However, the reason for this difference is an evolution of the experimental task design, given some difficulty from one of our early participants in comprehending the task instructions. The "memorable" or "not memorable" judgement was not universally understood; therefore, to avoid confusion in future patient testing, we shifted to the "like" or "dislike" judgement. However, it's important to note that all participants included in the present study clearly understood the task instructions and did not exhibit confusion regarding the orienting task, regardless of which version was used. We now include a statement on the nature of this difference in the Methods on page 13.

"Participants were asked to make a "like" or "dislike" judgment with respect to the image on each trial (or in the case of three of the 14 sessions a "memorable" or "not memorable" judgment) to orient and encourage participants to pay attention to each image. This difference is an evolution of the experimental task design given some participants noted confusion in comprehending the task instructions. However, all participants included in the present study confirmed a clear understanding of the task instructions regardless of which orienting task was used."

Minor Concerns

Reviewer 1 noted a minor typographical error on page 10, line 360 ("in the same the experiment").

RESPONSE: We thank the reviewer for this catch, and we have fixed this issue in several places throughout the manuscript.

Reviewer #2:

Reviewer #2 highlights the many strengths of the manuscript, including the use of causal manipulations of the amygdala-MTL circuit. The reviewer notes the study is strengthened by the results of content specificity for objects versus scenes and suggests this will be of high interest for memory researchers. They also note the experimental design and analyses were executed nicely and provide clean results that advance the literature on emotional memory, making the findings an important contribution to the field. The reviewer notes a few weaknesses, largely centered on some of the theoretical framing and interpretation of the findings, but suggests these concerns are addressable with revision.

1. Reviewer 2 noted that the proposed tension between the neuroscience and psychology literature that the present study is resolving seems superficial and needs to be situated in a more nuanced conceptual framework that is better supported by the empirical literature. The reviewer also requests more detail on the current state of long-axis differences in the MTL from the neuroimaging literature and suggests the inclusion of more background on the content specificity across the long-axis of the MTL.

RESPONSE: We appreciate the reviewer's comment. Our intent on illustrating the differences in the neuroscience versus psychology literature was to contextualize the direct versus largely indirect manipulations that have been used in each of these literatures, respectively. We aimed to highlight the *causal* manipulations of the amygdala from the neuroscience studies and contrast this with the largely *correlational* approaches used in psychology-inspired studies, to frame the present study as one that incorporates the methods and theory from *both* fields. However, we understand our approach may have created an artificial tension between the neuroscience and psychology literature that may not exist. We have changed the wording throughout the Introduction and Discussion sections to reduce the emphasis on "neuroscience vs. psychology" and more clearly articulate this difference as one of "direct vs. indirect" or "causal vs. correlational" manipulations.

We thank the reviewer for their perspective on the specificity of objects versus scenes and on the separation of information along the long axis of the hippocampus. We have provided more details regarding the anatomy and context for this separation in the Introduction on page 3. We have also incorporated the reviewer's suggested citation (Dolcos, Labar, and Cabeza, 2006) to provide more background on the role of the long-axis and emotional memory.

"Evidence from rats, humans, and non-human primates suggests that this anterior-to-posterior pattern of BLA connectivity parallels a functional trend of separation of non-spatial object information from that about spatial contexts[34–37]*. More specifically, a large body of literature indicates that the anterior hippocampus/perirhinal cortex is essential for recognition memory for individual items, such as images of objects*[38,39]*, whereas the posterior hippocampus mediates memory for images of places such as outdoor scenes*[40]*. The rodent hippocampus has a clear*

septal-to-temporal (posterior-to-anterior in primates) gradient for more to-less spatial information, and results from humans suggest a parallel gradient[35,41]. Moreover, although the relevant importance of the long-axis of the hippocampus is largely defined around anatomy, there is much parallelism from the lens of emotional memory studies in humans. For instance, activity in the amygdala and the MTL memory regions is strongly correlated and activity in anterior regions predicts memory for emotional items[42,43]. Consequently, the BLA appears to be anatomically situated to modulate memory for objects more so than memory for discrete scenes or spatial contexts."

2. Reviewer 2 requested an additional explanation for why theta-burst stimulation is not useful for probing network connectivity. The reviewer also asked whether connectivity can be measured during theta-burst stimulation and whether a phase coherence approach can be used, similar to what was done in our earlier paper (Inman et al., PNAS, 2018).

RESPONSE: We thank the reviewer for this comment. Studies suggest theta-burst stimulation is useful for modulating memory through the synchronization of neural activity, whereas single-pulse stimulation is the standard approach for assessing structural and effective connectivity. Given that one of the goals of the present study was to investigate BLA-MTL structural/effective connectivity, we employed a standard single-pulse evoked potential stimulation protocol. We agree that the nuances of this difference may not be obvious to our readers; we now include additional context for the use of single-pulse stimulation which can be found in the Introduction on page 4.

"Importantly, where theta-burst stimulation is useful for modulating memory through the synchronization of neural activity[21], using this stimulation pattern to probe simple network connectivity relationships is challenging in part due to confounding simultaneous stimulation artifacts. Single-pulse stimulation is the standard validated approach for assessing structural and effective connectivity[44]."

Unfortunately, stimulation artifact precludes electrophysiological analyses *during* electrical stimulation. The electrical artifact spreads across nearby channels and overwhelms the iEEG data at that time. Thus, we are unable to examine the effects of our theta-burst stimulation on BLA-MTL interactions during stimulation. Given the significant stimulation artifact, our earlier work (Inman et al., PNAS, 2018) examined phase coherence during memory *retrieval*, and not in direct association with the theta-burst stimulation that was delivered during encoding on the previous day. However, our collaborators are currently working towards making electrophysiological analyses during theta-burst stimulation possible through sophisticated stimulation artifact removal techniques (Xie et al., J Neural Eng, 2023). This is an area of ongoing work that we hope will make the analyses the reviewer is proposing possible in our future studies.

3. Reviewer 2 noted that the manuscript reports electrodes in the anterior and posterior hippocampus, however they are curious whether any electrode coverage exists for the PRC and PHC, given prior literature suggests these regions are important for object and scene memory, respectively.

RESPONSE: The reviewer raises an important limitation of the present study. Our analyses were restricted to where the electrodes were placed for clinical purposes; therefore, the subset of patients with electrodes simultaneously in the amygdala, aHC, pHC, PRC, and PHC is not sufficient for a properly powered analysis. We now include a statement that speaks to this limitation in the Discussion on page 11.

"Similarly, given our analyses were restricted to where electrodes were placed for clinical purposes, we were unable to sufficiently examine effective connectivity in the perirhinal cortex and parahippocampal cortex across all patients. Therefore, it remains unclear how these regions canonically associated with object and scene processing, respectively, respond to BLA stimulation."

4. Reviewer 2 expressed curiosity in how spatial proximity influences the effective connectivity measures and asked if regions closer to the simulation site always show greater connectivity.

RESPONSE: We appreciate the reviewer's comment. To account for the potential confound of spatial proximity between the stimulation site and the recording site, prior effective connectivity studies have implemented the use of bipolar re-referencing relative to nearest neighbor to account for volume conduction. Moreover, these studies have also excluded the ~10 ms prestimulation and poststimulation periods from analysis, given volume conduction effects and artefactual changes are largest during these periods. We implemented these same methods in the present study, and we now make this more explicit on page 13 in the Methods. Furthermore, there is consensus across many studies in the effective connectivity literature that show SPEP amplitude is not just a monotonic function of distance. That is, more distant sites can have larger evoked responses than closer ones, and work suggests SPEP amplitude is better predicted by white-matter tract properties than by simple Euclidean distance. Although we do not explicitly repeat these same analyses in the present manuscript, we do now address this point and cite the relevant literature in the Discussion on page 10.

"Specifically, single-pulse electrical stimulation of the BLA resulted in higher amplitude SPEPs in the aHC versus pHC which is consistent with our finding that direct electrical stimulation of the BLA enhances object memory but not scene memory. Moreover, these results are unlikely to be due to spatial proximity alone, as prior work suggests SPEP amplitude is not just a monotonic function of distance from the stimulation site[56–58]. Importantly, prior work suggests SPEP amplitude is better predicted by white-matter tract properties than by simple Euclidean distance[58]. That is, distance alone is unlikely to account for the network topographies we report."

5. Reviewer 2 and Reviewer 1 both noted the use of two different versions of the orienting task during encoding across participants, where for three of the 14 sessions a "memorable" or "not memorable" judgment was made with respect to the image on each trial, and in the case of 11 of the 14 sessions a "like" or "dislike" judgment. The reviewers request more clarity on this difference.

RESPONSE: We appreciate the reviewers' comments. An important note, as mentioned by Reviewer 2, is that although two different variations of the orienting task were used across the 14 encoding sessions, session is a random effect in our linear mixed effect model, thus this difference

across sessions is already accounted for at the level of our statistical testing (Dprime ~ 1 + Stim*ObjectScene + (1|Patient) + (1|Patient:Session)). However, the reason for this difference is an evolution of the experimental task design given some difficulty from one of our early participants in comprehending the task instructions. The "memorable" or "not memorable" judgement was not universally understood, therefore, to avoid confusion in future patient testing we shifted to the "like" or "dislike" judgement. However, it's important to note that all participants included in the present study clearly understood the task instructions and did not exhibit confusion with regard to the orienting task, regardless of which version was used. We now include a statement on the nature of this difference in the Methods on page 13.

6. Reviewer 2 suggests the negative findings of the stimulation awareness test do not appropriately control for this measure. They suggest incorporating the stimulation awareness data from Figure 3 into the multi-level modeling.

RESPONSE: We appreciate this suggestion and agree that this would be the most comprehensive way to incorporate the stimulation awareness data into our results. However, while there were 14 sessions of behavioral data in the manuscript, we were only able to conduct the stimulation awareness procedure in a subset of those patients (12 sessions). Several patients were explanted before we had an opportunity to conduct this test procedure, as we ran it only after all memory tests had been completed. Thus, given our already small sample size, it is difficult to determine whether we would observe the same overall effect in our linear mixed-effects model when we can include only a subset of the data. However, in our prior work (Inman et al., PNAS, 2018), a similar stimulation awareness procedure was conducted, yielding similar results of no subjective awareness of the stimulation. This suggests our memory-enhancement effect is unlikely to be due to subjective awareness of the stimulation. We now include a statement to this effect in the Results on page 7.

"Patients' responses indicate they were unable to reliably discriminate if or when any stimulation was being delivered (Fig. 3C) [$t_{(11)} = 1.24$, $p = 0.24$]. These results replicate those observed in prior work examining the effects of BLA stimulation on subjective awareness[20]."

7. Reviewer 2 suggests showing the amygdala stimulation sites across all patients on a single brain template to highlight the BLA region of interest.

RESPONSE: While we have already included all amygdala stimulation locations across participants on a single brain template in Figure 1B, we now also include the addition of a figure in the Supplemental Material that shows the stimulation electrodes in each individual patient for each session to preserve the patient-specific anatomy. See Supplementary Figure S5.

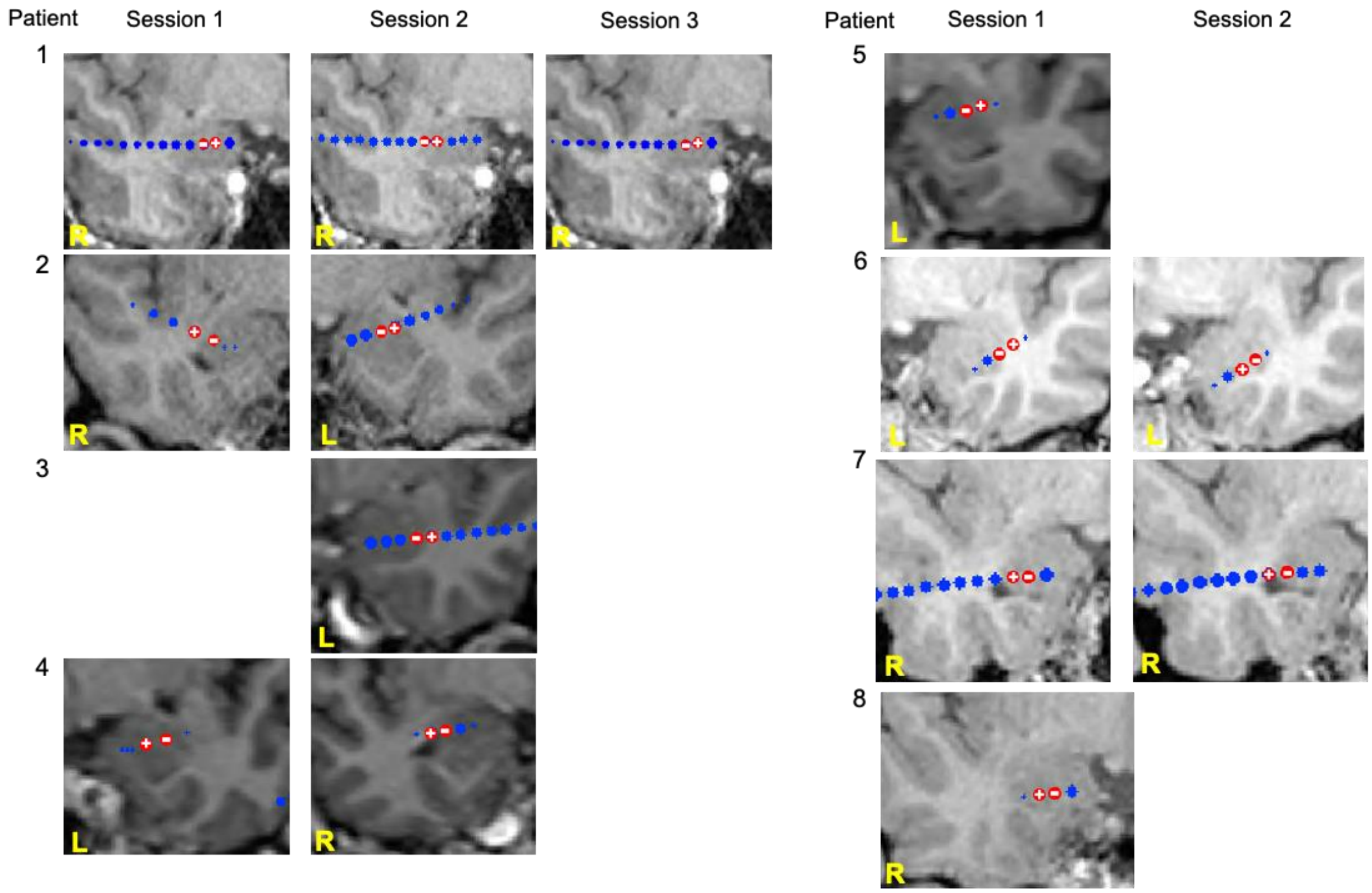

Supplementary Figure S5. Merged high-resolution preoperative MRI and postoperative CT (coronal view) in all 8 patients demonstrating the locations of the bipolar pair amygdala stimulation electrode contacts (in red) for all 14 stimulation sessions. Blue dots represent other electrode contacts along the same lead for reference. Electrodes were localized using LeGUI. Hemisphere stimulated: L, left; R, right. Anode/cathode electrodes: -, cathode; +, anode.

8. Reviewer 2 requested clarification on the core claim of selective memory enhancement for object memory but not scene memory when a significant interaction was not observed in the primary stimulation x stimulus-type analysis.

RESPONSE: We appreciate the reviewer's comment. Reviewer #1 had a similar comment. Please see our above response to Reviewer #1 (point 3).

9. Reviewer 2 suggested the addition of ROC curves for the confidence ratings data.

RESPONSE: We have now included ROC curves for the certainty judgments for objects and scenes for each session, which can be found in the Supplemental Material. See Supplementary Figure S7. We have also provided a description of the ROC results in the main manuscript text in the Results on page 7.

"Supplementary Fig. S7 shows the receiver operating characteristic (ROC) curves for stimulated and non-stimulated object and scenes images. The probability of correctly recognizing an old image (Hit) and the probability of incorrectly identifying a new image as a target (False Alarm) are plotted across varied certainty judgements for each patient/session. Area under curve (AUC) is a neutral measure of an ROC and a reliable measure of memory performance. Our results illustrate significantly higher AUCs for stimulated object images compared to non-stimulated

object images [$F_{(1,26)}$ = 10.71, p = 0.0030]. Stimulation did not affect AUCs for scene images [$F_{(1,26)}$ = 0.09, p = 0.77]. These results are consistent with those shown in Figure 2 and further suggest BLA stimulation enhances overall memory accuracy for object images."

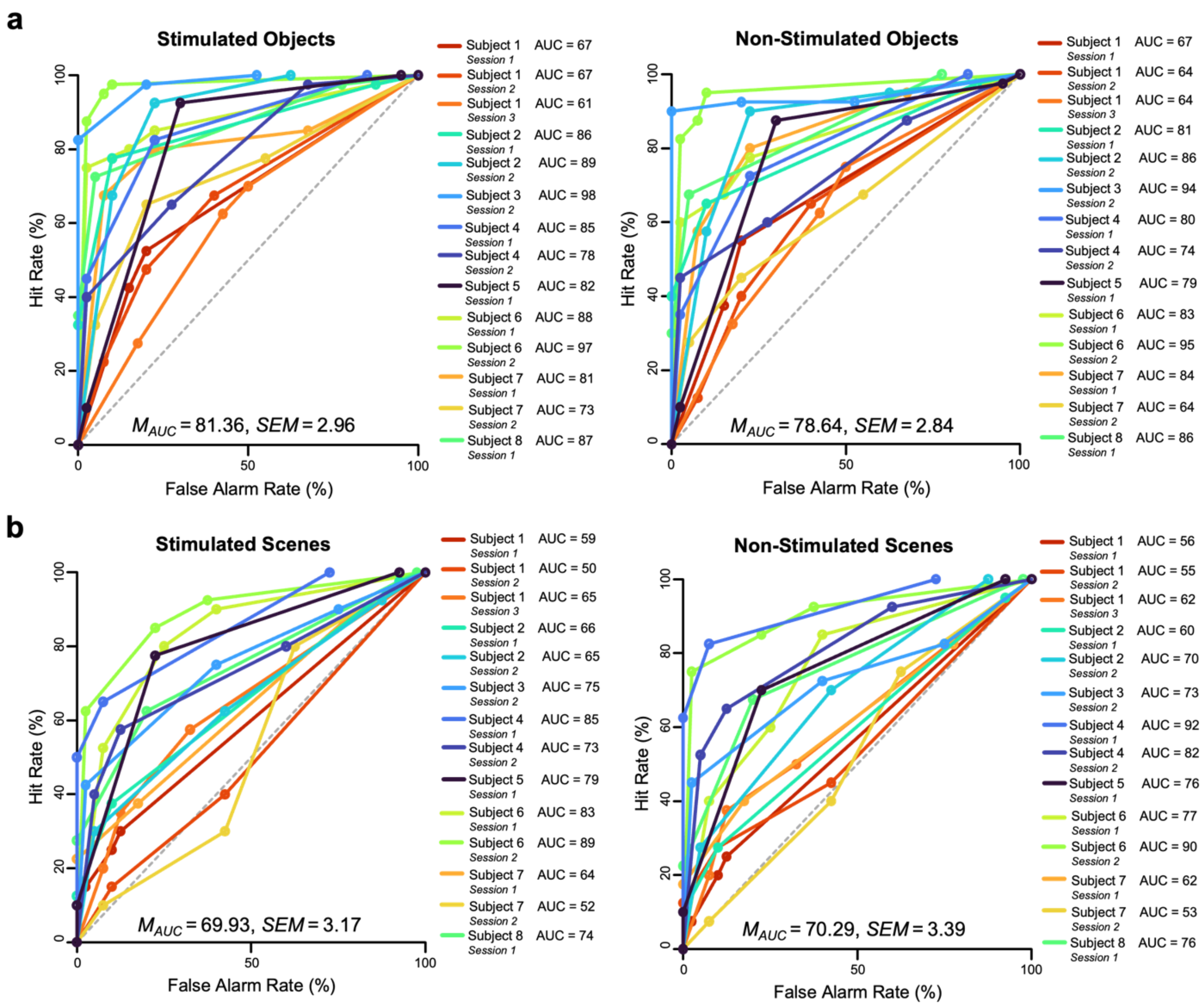

Supplementary Figure S7. Receiver Operating Characteristic (ROC) Curves. a, Stimulated object (left) and non-stimulated object (right) image discriminations for each of the 14 recognition-memory test sessions across eight patients. Probability of correctly recognizing an old object image (hit) vs. the probability of incorrectly identifying a new object image as a target (false alarm) across varied confidence ratings. **b**, Stimulated scene (left) and non-stimulated scene (right) image discriminations for each of the 14 recognition-memory test sessions across eight patients. Probability of correctly recognizing an old scene image (hit) vs. the probability of incorrectly identifying a new scene image as a target (false alarm) across varied confidence ratings.

10. All three reviewers were curious about any potential correlation between aHC-pHC effective connectivity differences and the memory findings.

RESPONSE: While we agree with the reviewers that our sample size is too small for statistical testing of a correlation between the effective connectivity results and the memory enhancement effects, we have provided a visualization of this correlation below as requested by the reviewers.

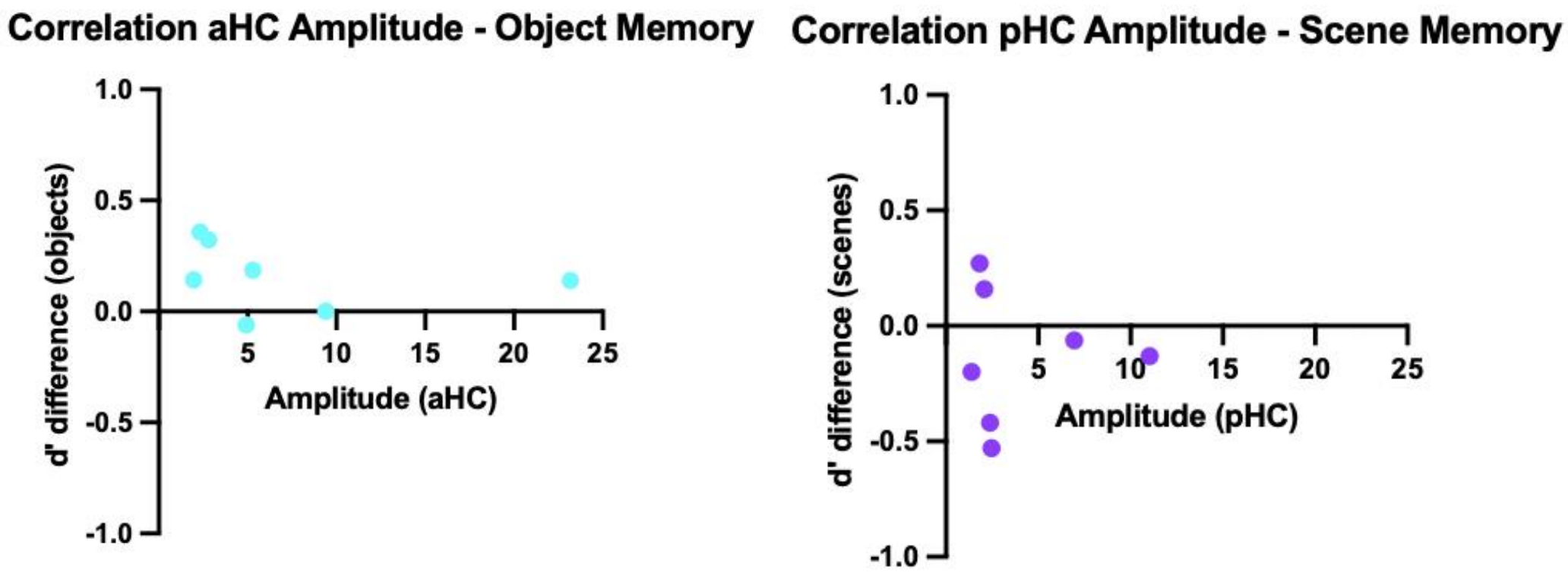

The anterior hippocampus (left) and posterior hippocampus (right) evoked response amplitudes from Figure 4C plotted against the recognition-memory performance scores (differences in d' in the stimulation and no-stimulation conditions) from Figure 2B for object and scenes, respectively.

<u>Reviewer #3:</u>

Reviewer #3 notes several strengths of the manuscript, including the important findings of the specificity of the amygdala's neuromodulatory influences on memory. The reviewer notes that the figures and supplemental information are appropriate. The reviewer had some criticisms, largely centered on missing details in the Methods section and the indirect link between BLA-hippocampal connectivity and memory performance. The reviewer suggests these concerns are addressable with revision.

1. Reviewer 3 requested clarity on whether the object and scene images were intermixed randomly during encoding and suggested including additional details on whether the number of stimulation trials was balanced across object and scene images.

RESPONSE: We appreciate these suggestions and confirm that object and scene stimuli were randomly intermixed during encoding and there were equal numbers of stimulated and non-stimulated object and scene images. We have provided more clarity on the experimental design in the Methods on page 13.

"During the encoding phase, each participant viewed 160 object and scene images (80 neutral object images and 80 outdoor scene images) one trial at a time (Fig. 1C) (see Supplemental material for additional details on the stimuli). The object and scene stimuli were randomly intermixed."

"Each image was presented for 3 s, and for a randomly selected half of the trials (stimulation condition), an offset of the image coincided with the onset of 1 s of stimulation to the BLA. Our randomization ensured there were equal numbers of object and scene images that were paired with stimulation and no stimulation."

2. Reviewer 3 noted inconsistencies with the number of encoding trials reported in the main manuscript and the supplemental material and suggested we provide clarity on whether the scrambled-only images were included across all sessions and whether they were ever paired with stimulation.

RESPONSE: We thank the reviewer for this comment. We have changed the wording in the Methods section on page 13 to maintain consistency across the manuscript. Additionally, we provide more clarity on the nature of the scrambled-only images which can be found on page 2 in the Supplemental Material.

"Object images were centered onto a subset of these scrambled backgrounds, and the remaining scrambled backgrounds were used as scrambled-only images (for controls in future electrophysiological analyses not within the scope of the present study). The scrambled-only images were never paired with amygdala stimulation and were randomly intermixed throughout the encoding phase but not presented at the retrieval test."

3. Reviewer 3 suggested it would be useful to determine whether the improvement in object memory depends on baseline memory performance under the no-stimulation condition. The reviewer also suggested a correlation between object memory and scene memory to determine whether patients showing greater improvements in object memory also tend to show stimulation effects for scene memory.

RESPONSE: We appreciate the reviewer's interest in these questions. We have now included a correlation between the stimulation-mediated object memory effects and the stimulation-mediated scene memory effects which can be found in the Supplemental Material. See Supplementary Figure S8. We have also provided a description of these results in the main manuscript text in the Results on page 6. In summary, a Pearson correlation revealed no significant linear association between the stimulation-mediated object memory effect and the stimulation-mediated scene memory effect, r(12) = .11, p = .72, 95% CI [–.45, .60]. The effect size was very small ($R^2 = .01$), indicating that X explained little variance in Y.

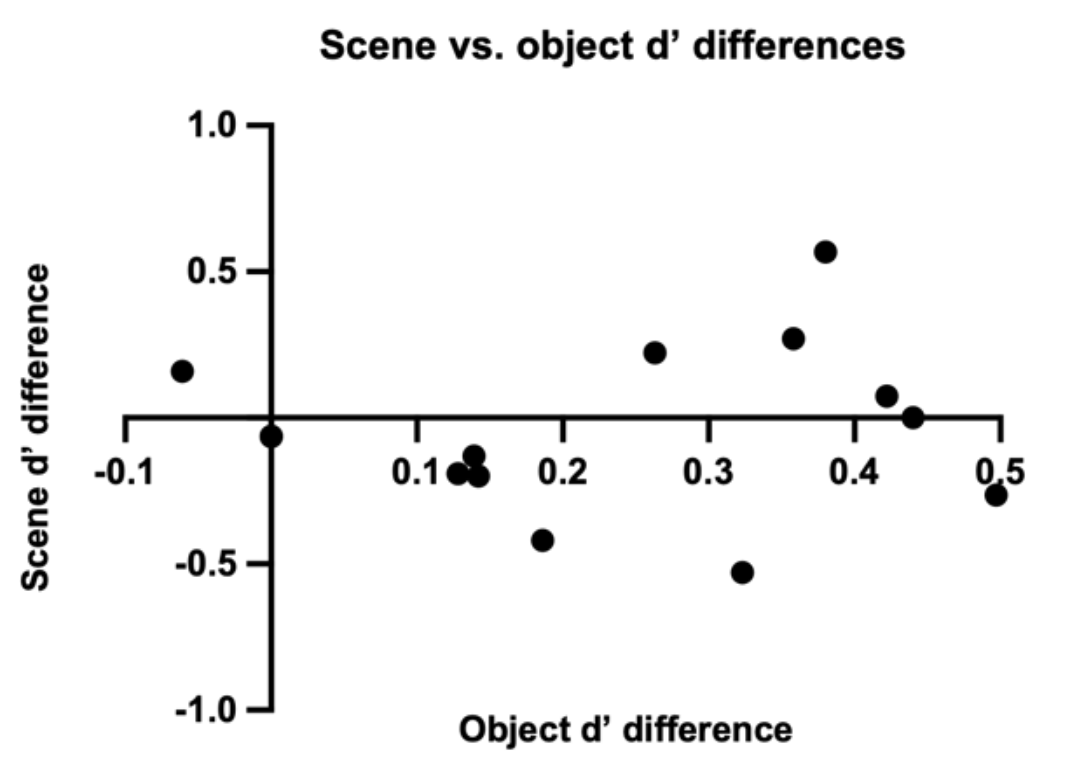

Supplementary Figure S8. Correlation between stimulation-mediated object and scene memory effects. Recognition-memory performance plotted for each session as the difference in d' in the stimulation and no-stimulation conditions for objects and scenes. There is no significant correlation between stimulation-mediated object memory effects and stimulation-mediated scene memory effects.

We have also provided a visualization below of the relationship between baseline memory performance under the no-stimulation condition and the stimulation-mediated memory effect for object images. Using simple linear regression, the results suggest a weak predictive relationship between baseline memory and the stimulation-mediated memory effect. The R squared value is 0.126, thus only 12.6% of the variance in the stimulation-mediated memory effect is explained by baseline memory. Although the slope is slightly positive (0.1016), the p value is not significant (0.213). Given this is a marginal correlation with few participants this analysis is limited in its informativeness by low statistical power, thus we have chosen not to include this information in the manuscript.

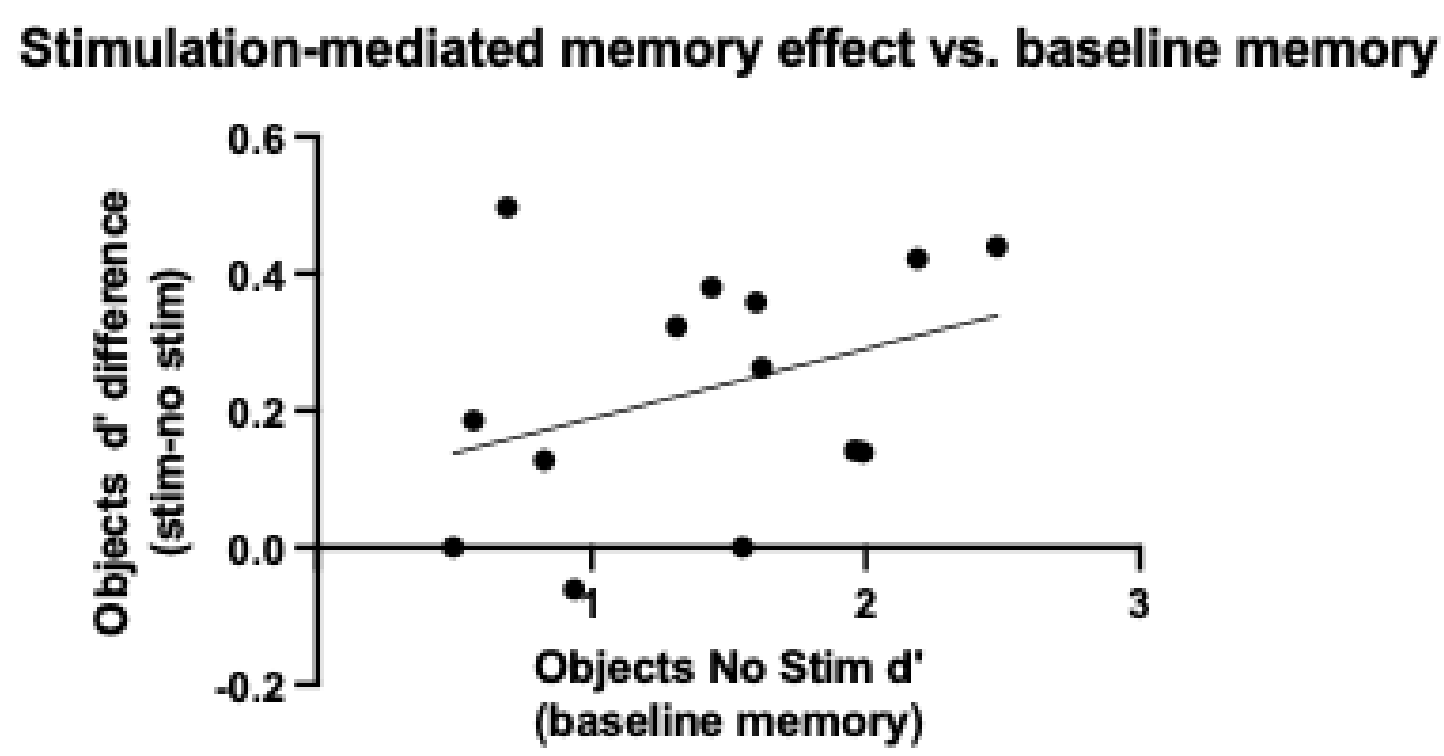

4. Reviewer 3 encouraged an analysis of hippocampal activity during encoding for object vs. scene-viewing to support the assumption that the aHC networks preferentially support object memory whereas pHC networks preferentially support scene memory.

RESPONSE: We thank the reviewer for this suggestion. We now more clearly define the large body of literature supporting the claim that the anterior MTL/perirhinal cortex is essential for recognition memory for objects, whereas the posterior MTL/parahippocampal cortex mediates memory for places/scenes. This can be found on page 3 of the Introduction. We agree that this analysis would be interesting, and we are currently pursuing it in relation to another manuscript in preparation exploring ERPs in the anterior vs. posterior MTL and how these ERPs during encoding correspond to subsequent memory for objects vs. scenes during memory retrieval. We look forward to incorporating the reviewer's suggestion into our ongoing analyses for this future manuscript.

"Evidence from rats, humans, and non-human primates suggests that this anterior-to-posterior pattern of BLA connectivity parallels a functional trend of separation of non-spatial object information from that about spatial contexts[34–37]. More specifically, a large body of literature indicates that the anterior hippocampus/perirhinal cortex is essential for recognition memory for individual items, such as images of objects[38,39], whereas the posterior hippocampus mediates memory for images of places such as outdoor scenes[40]. The rodent hippocampus has a clear septal-to-temporal (posterior-to-anterior in primates) gradient for more to-less spatial information, and results from humans suggest a parallel gradient[35,41]. Moreover, although the

relevant importance of the long-axis of the hippocampus is largely defined around anatomy, there is much parallelism from the lens of emotional memory studies in humans. For instance, activity in the amygdala and the MTL memory regions is strongly correlated and activity in anterior regions predicts memory for emotional items[42,43]. Consequently, the BLA appears to be anatomically situated to modulate memory for objects more so than memory for discrete scenes or spatial contexts."

5. All three reviewers were curious about any potential correlation between aHC-pHC effective connectivity differences and the memory findings. While the reviewers agree our sample size is too small for statistical testing of a correlation between the effective connectivity results and the memory enhancement effects, they requested a simple visualization of this relationship in the response to the reviews.

RESPONSE: We have provided a visualization of this correlation below as requested by the reviewers.

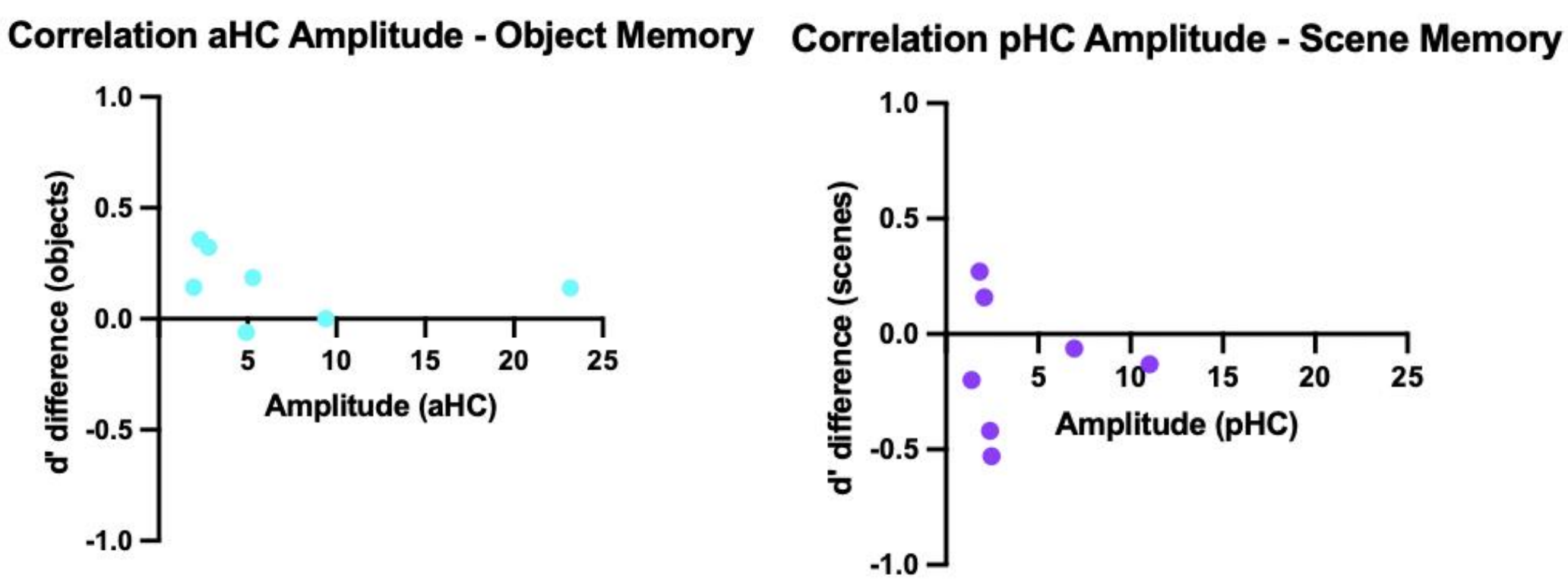

The anterior hippocampus (left) and posterior hippocampus (right) evoked response amplitudes from Figure 4C plotted against the recognition-memory performance scores (differences in d' in the stimulation and no-stimulation conditions) from Figure 2B for object and scenes, respectively.

6. Reviewer 3 noted that previous rodent studies demonstrate a role for the BLA in spatial/contextual learning, whereas the present results showed BLA stimulation did not influence scene memory. The reviewer suggested we comment on whether species differences, task demands, or stimulation parameters might account for this discrepancy.

RESPONSE: We thank the reviewer for this comment. Evidence from the primate brain indicates the BLA disproportionately projects to the anterior compared to posterior MTL, regions that process information about objects and scenes, respectively (see Wang & Barbas, 2018). Thus, the *human* BLA is anatomically situated to modulate memory for objects moreso than spatial contexts. The rodent MTL anatomy is slightly different (see Pikkarainen et al., 1999), which may contribute to the observed differences. Moreover, rodent work targeting the BLA itself shows enhanced spatial memory but also shows engagement of the dorsal hippocampus (posterior hippocampus in humans), important for spatial memory (see Wahlstrom et al., 2018). Whereas the present study shows stronger effective connectivity between the BLA and anterior compared to posterior hippocampus, suggesting our BLA stimulation is engaging the object memory system more than the spatial/contextual memory system. Finally, unlike the present experiment in which object and

scene memory were tested in the same paradigm, to our knowledge, no rodent study to date has tested multiple memory types in a single experimental paradigm. Thus, task demands and interactions between memory systems may be contributing to the present results. Nonetheless, we appreciate the reviewer's comment, and we now discuss these points in more depth in the Discussion on page 11.

"Finally, the observed memory-enhancement effect for objects was reliable and significant with no effect on scene memory, in contrast to previous studies that implicated the BLA in spatial and contextual learning. Evidence in rodents suggests optogenetic stimulation of the BLA enhances spatial memory and engages the dorsal hippocampus[18,55]. However, this prior work targeted BLA projections to the medial entorhinal cortex, suggesting that pathway manipulations are important for BLA modulation of spatial memory. Although we are unable to deliver electrical stimulation to distinct BLA projections in the human brain, our stimulation more fully engages the widespread projections of the BLA, and our results suggest stimulation preferentially engages the denser anatomical projections from the BLA to the aHC object memory system. Furthermore, unlike the present experiment in which object and scene memory were tested in the same paradigm, to our knowledge, no human or rodent study to date has directly compared multiple memory types in a single experimental paradigm. Thus, task demands and interactions between memory systems may be contributing to the present results."

7. Reviewer 3 expressed an interest in whether the memory-enhancing effects of BLA stimulation varied across stimulation hemisphere (right vs. left BLA) and location (medial vs. lateral BLA).

RESPONSE: Linear mixed-effects models predicting the effects of stimulation hemisphere (left/right BLA) and stimulation location (lateral/medial BLA) on recognition memory accuracy (d') revealed a significant main effect of stimulation hemisphere and a significant stimulation × hemisphere interaction. Specifically, memory performance was higher for sessions in which the left hemisphere BLA was stimulated compared to the right hemisphere BLA ($\beta = 0.59$, $p = .036$), and the effect of stimulation compared to no stimulation differed by hemisphere ($\beta = 0.17$, $p = .046$). Furthermore, there was a significant main effect of stimulation location and a trend towards a significant stimulation × location interaction. Specifically, memory performance was higher for sessions in which the lateral BLA was stimulated compared to the medial BLA ($\beta = -0.53$, $p = .007$). The interaction between stimulation condition and location did not reach significance ($\beta = 0.15$, $p = .077$). Together, these models indicate that stimulation-related effects on object memory performance are modulated by hemisphere but not robustly by BLA location. However, given our small sample size we are cautious in our interpretation of these results, and follow-up work should more thoroughly examine the effects of stimulation hemisphere and location on memory performance. Nonetheless, we have now included a figure in the supplemental material (Fig. S6) that illustrates the relationship between stimulation hemisphere and location, and supplemental text on page 3 that clarifies these results.

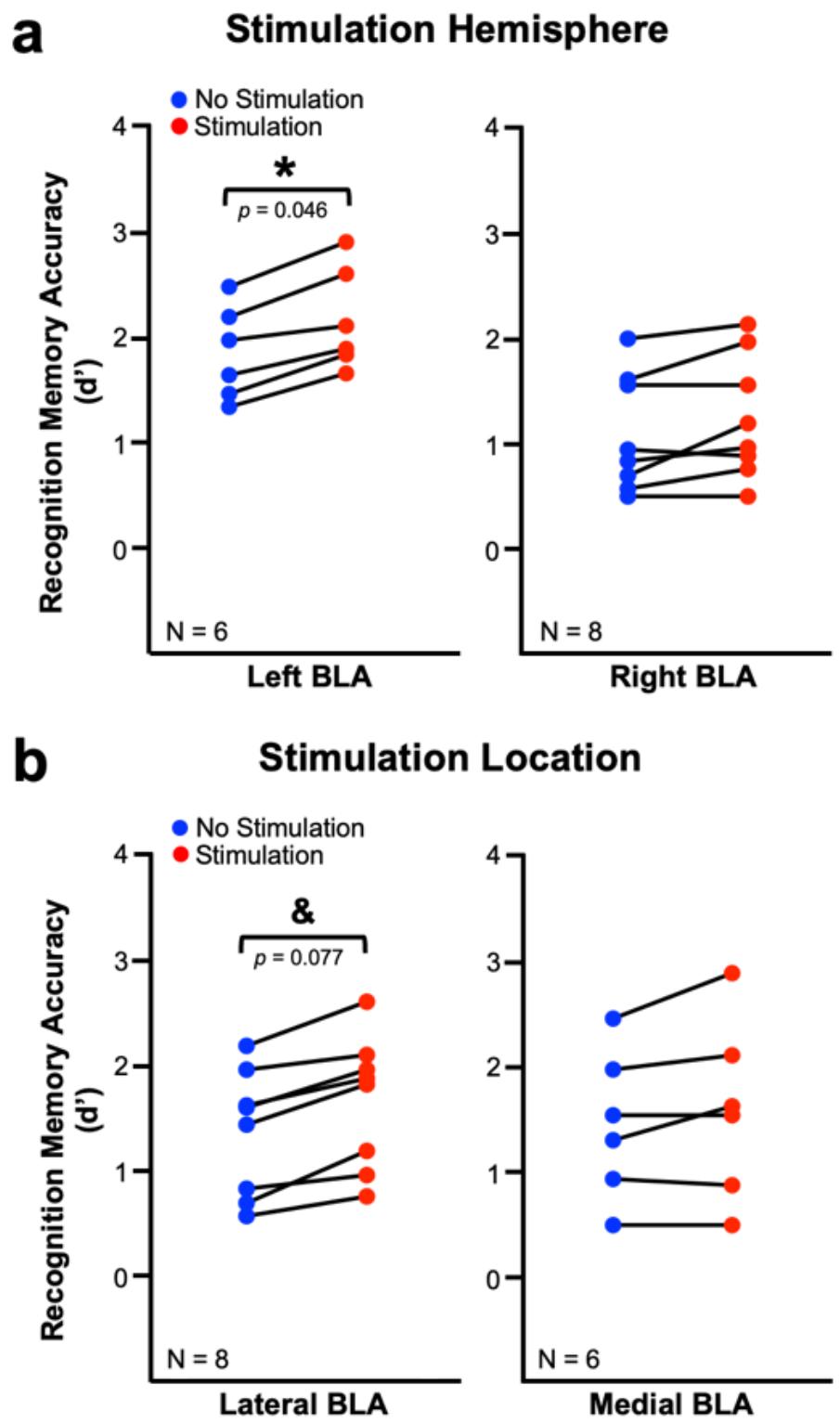

Supplementary Figure S6. Object Recognition Memory Performance Separated by Hemisphere and Location. **a**, Recognition-memory performance for objects for each of the 14 sessions across eight patients plotted as discriminability index (d') for left and right stimulation hemisphere sites. **b**, Recognition-memory performance for objects for each of the 14 sessions across eight patients plotted as discriminability index (d') for lateral and medial BLA stimulation sites. $*p < 0.05$; $\&p < 0.10$. Stimulation-related effects on memory performance are modulated by stimulation hemisphere but not robustly by BLA location.

Minor Concerns

Reviewer 3 noted inconsistent uppercase and lowercase panel labels in the supplementary figures compared to the figure captions.

RESPONSE: We thank the reviewer for this catch! We have now ensured all figure panel labels and figure captions in both the main manuscript and the supplementary text have lowercase lettering.

<u>Reviewer #4:</u>

Reviewer #4 co-reviewed this manuscript with one of the reviewers who provided the listed reports. We thank this reviewer for their efforts and Communications Biology for their initiative to facilitate training in peer review.

We thank the reviewers again for their time and efforts in providing another review of our manuscript. Based on their remaining concerns, we have revised the manuscript accordingly.

We have listed each reviewer's critique below. We also include direct quotes from revisions made to the main manuscript which can be found below in red.

Reviewers' Comments

Reviewer #1:

I would like to thank the authors for the careful revision. The manuscript has clearly improved, particularly in the clarification of the methods, the additional analyses, and the more explicit discussion of limitations. I also think the overall question—whether BLA stimulation differentially modulates distinct types of memory—is both important and well-motivated, and the combination of intracranial stimulation with behavioral and connectivity measures is a real strength of the work.

I only have a few remaining comments, mostly related to interpretation and phrasing.

1. Framing of stimulus-specific (object vs. scene) effects
The enhancement of object memory under stimulation is convincing and nicely demonstrated. At the same time, as the authors acknowledge, the primary stimulation × stimulus-type interaction was not significant.

While I understand the rationale for focusing on the planned contrast, I think the current wording (e.g., "selective" or "prioritized" enhancement) may be a bit stronger than what the data can fully support. To me, the results are clearly consistent with a preferential effect on object memory, but do not yet definitively establish selectivity.
I would suggest slightly softening the language here to better match the statistical evidence.

RESPONSE: We appreciate the reviewer's comment and agree that our results cannot definitively establish selectivity for objects over scenes. Thus, we have changed the wording throughout the manuscript to better match the statistical evidence.

Introduction page 5:

"These results are the first to use direct brain stimulation and measures of effective connectivity to provide a potential circuit mechanism by which the amygdala preferentially enhances object memory compared to scene memory in humans."

Discussion page 9:

“The present study suggests that a potential circuit mechanism by which BLA stimulation preferentially enhances object memory compared to scene memory involves increased effective connectivity between the BLA and aHC, compared to the BLA and pHC.”

Discussion page 10:

“To further understand the BLA’s preferential effects on object learning, it is also critical to investigate the direct relationship between the areas stimulated and the resulting behavioral effect.”

Conclusions page 12:

“Our findings suggest the BLA mediates an important preference for what information is prioritized in memory, rather than a general enhancement of all memory, and that BLA interactions with the hippocampus may be important for this preference.”

2. Interpretation of effective connectivity results
The addition of the connectivity analysis is a nice extension and adds an interesting mechanistic angle to the paper. That said, I would still interpret this part somewhat cautiously.

As presented, these analyses are based on a smaller subset and are not directly linked to the behavioral effects. For that reason, it may be more appropriate to frame them as pointing to a possible or candidate circuit mechanism, rather than a definitive one.

RESPONSE: We appreciate the reviewer’s comment, and we have changed the wording throughout the manuscript to reflect a *potential* circuit mechanism, rather than a definitive one.

Introduction page 5:

“These results are the first to use direct brain stimulation and measures of effective connectivity to provide a potential circuit mechanism by which the amygdala preferentially enhances object memory compared to scene memory in humans.”

Results page 9:

“Together, these results suggest stronger effective connectivity between the BLA and aHC compared to the BLA and pHC, providing a potential mechanism by which BLA stimulation enhances object memory over scene memory.”

Discussion page 9:

"The present study suggests that a possible circuit mechanism by which BLA stimulation preferentially enhances object memory compared to scene memory involves increased effective connectivity between the BLA and aHC, compared to the BLA and pHC."

Conclusions page 12:

"Furthermore, single pulse electrical stimulation of the BLA elicits higher magnitude evoked responses in the aHC compared to the pHC, suggesting a potential mechanism by which BLA stimulation enhances object memory but not scene memory."

Reviewer #2:

I thank the authors for their thoughtful reviews, and only have one minor concern. The authors note that they do not show an interaction, but they can use difference scores, due to the small sample size and multi-level modeling approach. I completely agree with the authors rationale, but I think some discussion of this limitation in statistical analyses should be addressed in the manuscript, if only to inform readers of their analytical approach.

RESPONSE: We appreciate the reviewer's comment and agree that transparency regarding our statistical approach is important. We have now included a paragraph in the Discussion section on page 11 that details our approach and its limitations.

*"Second, while our results indicate an effect for BLA stimulation on object memory but not scene memory a standard 2 x 2 model did not yield a significant interaction for objects and scenes. Our linear mixed effects model accounts for the tangential random effects variability of patients and sessions (Dprime ~ 1 + Stim*ObjectScene + (1|Patient) + (1|Patient:Session)). However, given the constraints of collecting data from relatively rare patients in a clinical setting, our dataset is small, necessitating relatively few model parameters. Thus, with the present statistical approach we are unable to account for the random effects of stimulation on object and scene memory that might vary across patients and/or sessions because the model will not converge on a viable solution for this most fully parameterized model (Dprime ~ 1 + Stim*ObjectScene + (Stim*ObjectScene|Patient) + (Stim*ObjectScene|Patient:Session)). More specifically, the resulting covariance matrix is not positive definite, due to more model parameters than the data can support. However, in principle, a 2 x 2 interaction is a difference of differences. Thus, the significant result in Figure 2B showing the recognition-memory performance plotted for each session as the difference in d' between the stimulation and no-stimulation conditions for objects*

and scenes more explicitly illustrates the magnitude of the amygdala-mediated memory enhancement for objects versus scenes."
